# Yeast Phenomic Analysis Reveals DNA Repair, pH Homeostasis, and Ribosomal Biogenesis as Modulators of Anticancer Ruthenium Complex KP1019

**DOI:** 10.3390/ijms27073275

**Published:** 2026-04-04

**Authors:** Amanda F. Bible, Jackson S. Blackman, John W. Rodgers, Samuel R. Gary, Megan Rainey, Mary E. Miller, Alexander Stepanov, John L. Hartman 4th, Laura K. Stultz, Pamela K. Hanson

**Affiliations:** 1 Cognitive Neurology, Ochsner Medical Center, New Orleans, LA 70121, USA; 2 Department of Neurobiology and Behavior, University of California Irvine, Irvine, CA 92697, USA; jsblackm@uci.edu; 3 Department of Genetics, University of Alabama at Birmingham, Birmingham, AL 35294, USAastepano@uab.edu (A.S.); jhartman@uab.edu (J.L.H.4th); 4 School of Medicine, University of Colorado, Aurora, CO 80045, USA; samuel.gary@cuanschutz.edu; 5 Department of Biology, Rhodes College, Memphis, TN 38112, USAmillerm@rhodes.edu (M.E.M.); 6 Heersink School of Medicine, University of Alabama at Birmingham, Birmingham, AL 35294, USA; 7 Department of Biology, Furman University, Greenville, SC 29613, USA; 8 Department of Clinical and Diagnostic Sciences, University of Alabama at Birmingham, Birmingham, AL 35294, USA

**Keywords:** KP1019, anticancer ruthenium complex, *Saccharomyces cerevisiae*, high throughput cell phenotyping, DNA damage response, ribosomal biogenesis

## Abstract

The anticancer ruthenium complex indazolium *trans*-[tetrachlorobis(1*H*-indazole) ruthenate (III)—also known as KP1019—inhibits cancer cell proliferation in vitro, causes tumor regression in animal models, and showed no dose-limiting toxicity in a phase I clinical trial. Previous studies found that KP1019 damages DNA in both cancer cells and the budding yeast *Saccharomyces cerevisiae*. To identify other potential targets of KP1019 along with pathways that modulate the drug’s cellular effects, we screened the yeast gene deletion strain library by quantitative high-throughput cell array phenotyping (Q-HTCP). Fitness differences, as judged by growth curve analysis, identified genes for which loss of function (gene deletion) interacts with (enhances or suppresses) KP1019 effects. Drug-enhancing deletions were enriched for DNA repair functions, consistent with DNA damage being a primary target of KP1019 in yeast. pH homeostasis also modified the effects of KP1019. Drug-suppressing deletions prominently involved ribosomal proteins. A mechanistic link between ribosomal protein function and KP1019 toxicity was supported by dose-dependent accumulation of Rpl7a-GFP in the nucleolus, which is a hallmark of ribosomal biogenesis stress. Furthermore, KP1019 acted synergistically with the TOR pathway inhibitor everolimus to inhibit cell proliferation. The resulting model, wherein KP1019 perturbs ribosome assembly, can inform the design of future combination therapies.

## 1. Introduction

Thoroughly understanding the mechanism of an anticancer drug, as well as its off-target effects, has the potential to improve patient outcomes by enabling personalized medicine and driving the design of combination therapies. However, detailed, unbiased mapping of drug mechanisms remains a significant challenge. Genetic screens in the budding yeast *Saccharomyces cerevisiae* can serve as a valuable tool in clarifying drug mechanisms and identifying modulators of drug sensitivity. For example, a mutagenesis-based screen helped discover the role of *S. cerevisiae* Ctr1 and its murine ortholog in cisplatin uptake [1]. Subsequent studies verified the role of human Ctr1 in the uptake of platinum-based drugs in a variety of cancers [2].

Construction of the yeast deletion collection has enabled a wide range of pharmacogenomic screening strategies [3], including quantitative phenomics [4,5]. When this approach is applied, the over 4000 strains in the collection, each of which is missing a different non-essential gene, can be assayed under multiple media conditions (e.g., escalating drug concentrations) in a single experiment. Time-lapse imaging generates rich data for growth curve analysis. The fitted data for each growth curve for each strain under each condition provides growth curve parameters, including the carrying capacity, *K*; the maximum specific growth rate, *r*; and the time required to reach half carrying capacity, *L* [4,6]. Each parameter can be considered a distinct phenotype. These quantitative phenotypes can uncover interactions between genes and the environment. For example, this strategy elucidated how carbon metabolism can alter which networks of genes modulate sensitivity to the chemotherapeutic drug doxorubicin [7].

**Figure 1:**
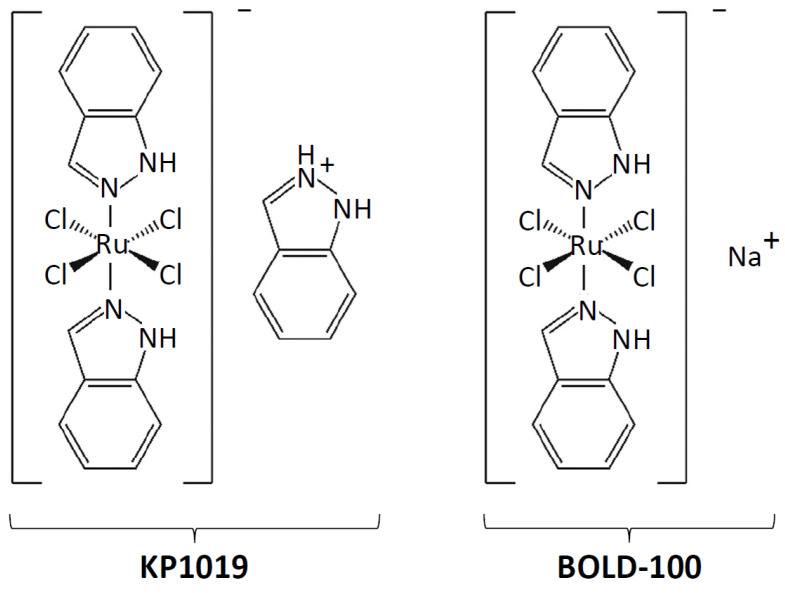
Structures of KP1019 and its sodium salt analog BOLD-100.

Here, we present findings from a yeast phenomic study of indazolium *trans*-[tetrachlorobis(1*H*-indazole)ruthenate(III)], also known as KP1019 (Figure 1). This anticancer-ruthenium complex inhibits the proliferation of cells from diverse explanted human tumors [8]; triggers apoptosis in colorectal, hepatocellular, and cervical carcinoma cell lines in vitro [9,10]; causes regression of autochthonous tumors in rats [11]; and was well tolerated in a phase 1 clinical trial, stabilizing disease progression in five of six evaluable patients without dose-limiting toxicity [12,13]. Alongside its therapeutic promise, questions about the mechanism of action of KP1019 remain. Previous studies suggest that the drug causes oxidative stress and double-strand breaks in the DNA of colorectal cancer cells [9]. These aspects of the drug’s mechanism appear to be evolutionarily conserved since KP1019 also causes DNA damage and mild oxidative stress in yeast [14,15,16,17]. Additional targets and mechanistic details warrant further research. For example, the drug accumulates not only in the nucleus but also in the cytoplasm and mitochondria [10,18,19,20,21], yet the physiological significance of this localization pattern remains unclear.

Additional controversy surrounds both the mode by which KP1019 is internalized and the drug’s proposed activation by reduction of the central ruthenium atom from Ru(III) to Ru(II). Given the similarity of ruthenium to iron, it was hypothesized that ruthenium could be internalized by the transferrin pathway [22]. However, conflicting results were obtained when comparing the uptake of free vs. transferrin-bound KP1019 [23,24]. Moreover, the drug is readily internalized by the budding yeast *Saccharomyces cerevisiae*, which lacks the transferrin pathway [14,20]. With respect to drug activation, the reducing agent ascorbic acid can increase KP1019 toxicity as well as binding to DNA [25]. However, electron paramagnetic resonance studies in yeast and X-ray absorption spectroscopic studies of HepG2 cells showed that the central ruthenium atom of KP1019 remains in the Ru(III) state after uptake [20,21]. These later findings are inconsistent with the activation by reduction model and reopen questions regarding the mechanism of KP1019’s apparent selectivity for cancer cells.

Thus, the goal of this study is to use yeast phenomics to identify modulators of KP1019 sensitivity. Previously, Golla et al. used transcriptomic data to inform the selection of nearly 300 yeast deletion strains to examine them phenotypically [26]. However, the fitness of deletion strains correlates weakly with the regulation of the corresponding genes [27]. So, here, we report the first phenomic screen of the larger yeast deletion strain library. These results shed important light on previous observations in both yeast and cancer cells and may help explain previously documented synergies between drugs similar to KP1019 and other chemotherapeutic agents.

## 2. Results

### 2.1. Global Response of Yeast Deletion Collection to KP1019

**Figure 2:**
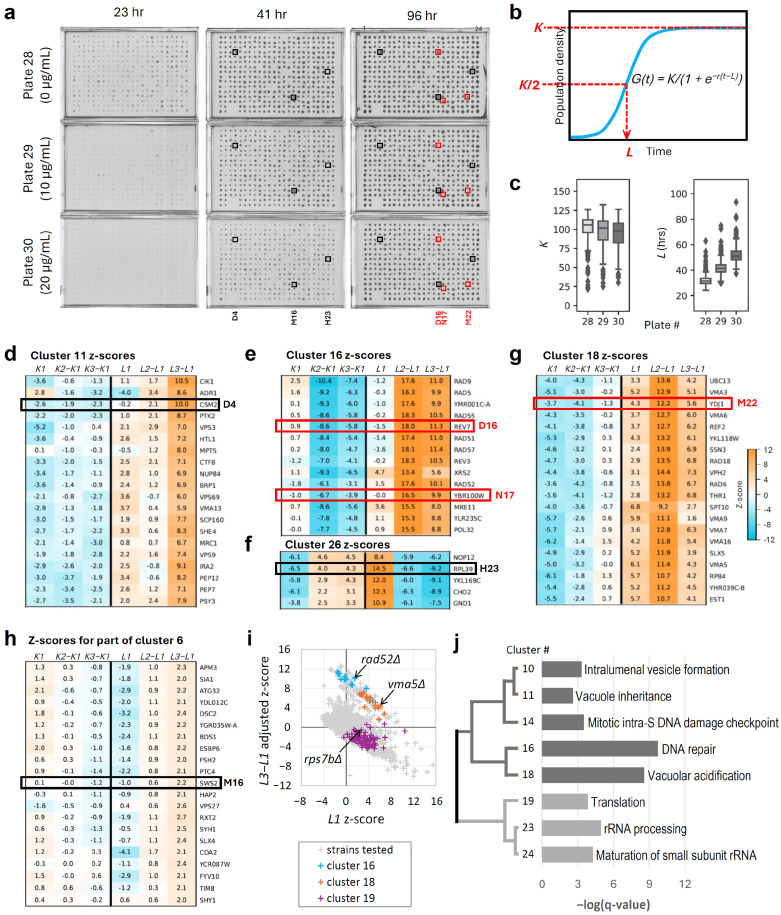
Quantitative phenotypic profiling to identify genetic modulators of KP1019 tolerance. (**a**) Images of an example 384-strain array spotted onto solid media containing 0, 10, and 20 µg/mL KP1019 and imaged at the same three timepoints. Highlighted spots correspond to strains featured in subsequent panels. Black boxes indicate strains highlighted at both 41 and 96 h. Red boxes indicate strains highlighted at 96 h only. (**b**) A theoretical growth curve is shown and can be described by the equation *G*(*t*) = *K*/(1 *+ e^−r(t−L)^*). Dashed lines represent the cell proliferation parameters *K* and *L*. Example growth curves are available in Supplemental File S2 (Figure S1). (**c**) Box plots showing *K* and *L* distributions for the cell array shown in panel (**a**). Box plots for all 16 cell arrays under all three treatments are available in Supplemental File S1. All data used to calculate *K* and *L* z-scores for the entire screen are available in Supplemental File S3. (**d**–**h**) Clustering based on z-scores reveals (**d**) weak to moderate drug-enhancing deletions, (**e**) strong drug-enhancing deletions, (**f**) drug-suppressing deletions, (**g**) intermediate drug-enhancing deletions, and (**h**) very weak drug-enhancing deletions. (**i**) Scatter plot depicting the relationship between adjusted *L3* − *L1* z-scores (a measure of resistance or sensitivity) and *L1* z-scores (a measure of fitness). (**j**) Dendrogram summarizing hierarchical clustering results. Dark gray lines and bars correspond to KP1019 sensitive clusters. Light gray lines and bars correspond to KP1019 resistant clusters. For brevity, only clusters gene ontology (GO) terms below a false discovery rate threshold (q < 0.005) are shown, along with the lowest q-value GO term for each respective cluster. Full clustering and GO term results are available in Supplemental Files S4 and S5.

To identify novel modulators of the cellular response to the anticancer ruthenium complex KP1019, we conducted an unbiased quantitative phenomic screen of the yeast gene deletion collection. As illustrated in Figure 2a, yeast deletion strains and wild-type controls were grown on defined media containing 0, 10, or 20 µg/mL KP1019. The agar cell arrays (plates) were scanned at regular intervals, and these data were subjected to image analysis and fitting to a logistic growth function, allowing determination of the cell proliferation parameters (CPPs) for each strain under each condition (Supplementary Files S1–S3). CPPs of interest include carrying capacity (maximum total growth, *K*), maximum growth rate (*r*), and time to half carrying capacity (*L*) (Figure 2b) [5,6,7]. Since *L* is a more precise CPP than *r*, likely because *r* occurs at very low population size and is thus not directly measurable with our imaging technology, subsequent analyses focused on *K* and *L* [6]. When considering these two CPPs, growth inhibitory drug concentrations are manifested by decreasing *K*, increasing *L*, or both (Figure 2c). The majority of outliers had *K* values below the interquartile range, *L* values above the interquartile range, or both, suggesting that the screen was particularly effective at identifying KP1019-enhancing gene deletions.

Since the effects of KP1019 varied somewhat across different plates (Supplemental File S1), the CPPs for each strain at each concentration of KP1019 were standardized as z-scores. Specifically, the median CPP for each plate was subtracted from the CPP for each deletion strain on that plate. To correct for assay variation, these values were then divided by the standard deviation of the CPP for the 384-spot array of the reference strain. The standardized data (z-scores) for each CPP allowed for comparisons between plates so that gene–drug interactions could be identified. The interaction model employed in this study expects the fitness of a strain in the presence of the drug to be proportional to the fitness of the same strain in the absence of the drug [28]. So, the CPP z-score of a strain in the presence of the drug is expected to equal the CPP z-score in the absence of the drug plus the interaction (*CPP_z_drug = CPP_z_nodrug + interaction*). Thus, the interaction itself will equal the CPP z-score of the strain in the absence of the drug, subtracted from the CPP z-score of the strain in the presence of the drug. This subtraction step (*CPP_z_drug − CPP_z_nodrug*) was used to generate adjusted z-scores as a measure of interaction between KP1019 and the gene deletion. Given the use of z-scores, which standardize the data for each 384-stain array and thus also each concentration of the drug, the expectation from this model is that the interaction value for most strains will be at or near zero. Deviation from this expectation can identify deletions that enhance or suppress the bioactivity of KP1019.

Figure 2d–h present the results of hierarchical clustering of adjusted z-scores alongside unperturbed (0 µg/mL KP1019; *L1* or *K1*) z-scores as a reference for growth variation due to the deletion alone. This visualization of the data reveals diverse patterns. For example, clustering distinguished gene deletions with strong interactions (high or low adjusted z-scores) with KP1019 (Figure 2e) from those with moderate interactions (Figure 2h). Distinct clusters also highlighted differential dose responses to KP1019. For instance, gene deletions in cluster 11 showed enhancement mostly at 20 µg/mL KP1019, whereas deletion strains in cluster 18 were also strongly impacted by 10 µg/mL KP1019 (Figure 2d,g, respectively), indicating greater sensitivity. Importantly, *K*- and *L*-adjusted z-scores were independently informative, as illustrated by the distinct patterns of different clusters (Figure 2d–h). In general, *K*-interactions identified gene deletions with the strongest drug-enhancing effects, while *L* revealed both intermediate enhancement as well as suppressing interactions. Thus, *K* and *L* are complementary and mutually informative. Gene deletions that cause intrinsic fitness defects (low *K* or high *L* z-scores in the absence of the drug) can show enhancement, suppression, or no effect on the drug response. This point is illustrated by Figure 2f,g, which features clusters of strains with relatively low *K* z-scores in the absence of KP1019. However, gene deletions in cluster 26 suppressed KP1019 activity (Figure 2f), while gene deletions in cluster 18 enhanced KP1019 activity (Figure 2g). The lack of strict correlation between strain fitness and drug modulation is also illustrated by Figure 2i, which shows that strains within drug-enhancing cluster 18 and drug-suppressing cluster 19 have substantial overlap in their range of *L1* z-scores. Note that spot H23 (*rpl39∆*) in Figure 2f is a suppressor. Similar to most suppressors in this study, *rpl39∆* had reduced fitness in the absence of the drug (*K1* z-score = −6.5; *L1* z-score = 14.5), but it is relatively non-responsive to KP1019, resulting in high adjusted *K* z-scores (4 and 4.3) and negative adjusted *L* z-scores (−6.6 and −9. 2) (Figure 2f; Supplementary File S2).

Genes within each cluster were subjected to gene ontology (GO) term enrichment analysis. Representative GO terms for clusters with significant enrichment are shown in Figure 2j. For brevity, the false discovery rate threshold q < 0.005 was used as a cut-off for inclusion in the figure. However, full clustering results and all GO terms for all clusters are available in Supplemental Files S4 and S5. The dendrogram depicts two primary clades. The upper clade corresponds to drug-enhancing deletions enriched for GO terms related to vacuolar inheritance and acidification, DNA repair, and the S-phase DNA damage checkpoint. The lower clade corresponds to clusters of strains with KP1019-suppressing deletions. GO terms for this clade relate to ribosomal biogenesis (ribi), structure, or function. Additional validation of the interaction between ribosomal protein (RP) deletion and KP1019 is provided in Supplemental File S2 (Figures S2 and S3).

Modulation of drug activity by DNA repair and ribosome function is further supported by the lists of strongest outliers. For example, within the list of the ten drug-enhancing deletions with the lowest adjusted *K* z-score at 10 µg/mL KP1019, eight have well-established roles in DNA repair (Table 1). Furthermore, within the list of the ten drug-suppressing deletions with the lowest adjusted z-score for *L* at 20 µg/mL KP1019, three are components of the ribosome, one overlaps with a mitochondrial RP gene, and one overlaps with a nucleolar protein gene required for ribi (Table 2).

### 2.2. KP1019 Damages DNA

**Figure 3:**
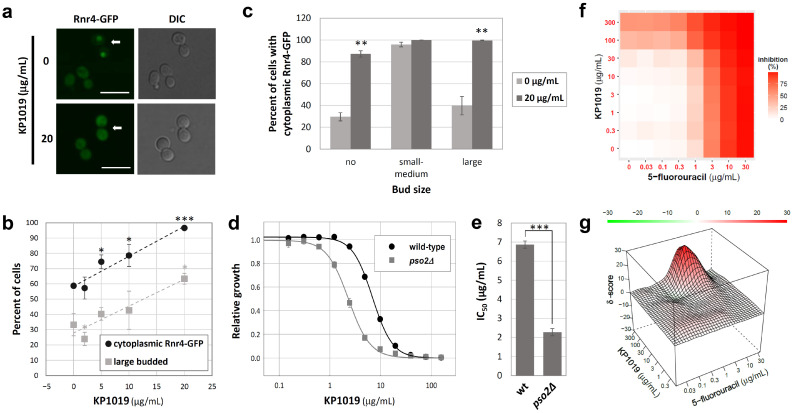
Verification of DNA as a target of KP1019. (**a**) Representative micrographs from Rnr4-GFP-expressing yeast treated with and without KP1019 for 3 h. Arrows indicate cells with large buds. Scale bar, 10 μm. (**b**) Bud size and Rnr4-GFP localization were scored for at least 250 cells per KP1019 concentration per trial. Data are the mean ± standard deviation for three biological replicates. Dashed lines correspond to linear regression, which revealed a strong relationship between drug concentration and bud size (R^2^ = 0.72, F(1, 13) = 33.82, *p* < 0.001) and between drug concentration and cytoplasmic Rnr4-GFP localization (R^2^ = 0.86, F(1, 13) = 77.75, *p* < 0.001). * *p* < 0.05, *** *p* < 0.001 for the same readout relative to no drug control, as determined by paired *t*-test. (**c**) Rnr4-GFP localization data were disaggregated based on bud size. Data presented are the mean and standard deviation for three biological replicates. ** *p* < 0.01 relative to no drug control as determined by paired *t*-test. (**d**,**e**) Yeast cells were grown overnight in varying concentrations of KP1019; then, the culture optical density was measured at 600 nm. Data presented are the mean and standard deviation of three trials. Four-parameter logistic curves were fit to the data for each trial to determine half-maximal inhibitory concentrations (IC_50_s). *** *p* < 0.0001 as determined by Student’s *t*-test (**f**,**g**). Exponentially growing wild-type yeast cells were treated with varying concentrations of KP1019 for 3 h prior to 18–24 h of growth in the presence of 5-FU. The response matrix (**f**) and interaction landscape (**g**) represent the average of three biological replicates. Results of individual trials are available in Supplemental File S2 (Figure S4).

Given that this phenomic screen implicated DNA as a probable target of KP1019 (Figure 2e,j), we verified that the concentrations of KP1019 used in this study were sufficient to activate the DNA damage response by monitoring the localization of GFP-tagged Rnr4. This protein is a subunit of the ribonucleotide reductase (RNR) complex that catalyzes the rate-limiting step of nucleotide synthesis, which is required for both DNA replication and repair [30,31]. Importantly, the Rnr2-Rnr4 heterodimer is a downstream effector of the DNA damage response in yeast, as activation of this signaling pathway triggers redistribution of Rnr2-Rnr4 from the nucleus to the cytoplasm, where the full RNR complex forms and synthesizes deoxyribonucleotides (dNTPs) [32,33]. As seen in Figure 3b, KP1019 caused a dose-dependent increase in the percentage of cells with cytoplasmic Rnr4-GFP (R^2^ = 0.86, F(1, 13) = 77.75, *p* < 0.001). This change in localization correlates with drug-induced mitotic arrest, indicated by the increased prevalence of yeast cells with large buds (Figure 3b) [14,15]. To verify that this change in localization was not an artifact of the impact of KP1019 on the cell cycle, Rnr4-GFP localization was also analyzed as a function of bud size. Although mutations can uncouple budding from replication, when wild-type yeast have small- to medium-sized buds, they are typically in the S-phase [34], which is supported by an increase in cytoplasmic localization of the Rnr2-Rnr4 heterodimer [32,35]. In contrast, Rnr4 is typically nuclear in mitotic yeast, as well as in unbudded G1 cells [32,35]. Similar trends were observed in our untreated controls, as cytoplasmic localization peaked in untreated cells with small- to medium-sized buds (Figure 3c). However, when yeast cells were treated with 20 µg/mL of KP1019, there was a dramatic and statistically significant (*p* < 0.01) increase in cytoplasmic localization of Rnr4-GFP in unbudded and large budded cells (Figure 3c), which supports the conclusion that KP1019 damages DNA.

Beyond implicating DNA damage generally, GO term analysis indicated that loss of translesion synthesis (TLS) and homologous recombination (HR) enhanced KP1019 toxicity (Supplemental File S5). This result is consistent with previous work [14], demonstrating the validity of the screen. The DNA damage checkpoint cluster (Figure 2j) was associated with multiple GO terms, twelve of which featured *PSO2*, a gene that encodes a highly conserved nuclease critical for interstrand cross-link (ICL) repair in yeast [36,37]. Since ICL repair was not among the GO terms uncovered by the screen, we measured the growth of *pso2Δ* yeast in a wide range of KP1019 concentrations. As seen in Figure 3d,e, yeast lacking *PSO2* had a lower half-maximal inhibitory concentration (IC_50_) than the wild-type control (2.3 ± 0.2 vs. 6.9 ± 0.2 µg/mL, *p* < 0.0001), indicating a significant increase in KP1019 sensitivity, which further validated the screen.

Given that the TLS, HR, and ICL DNA repair pathways involve synthesis of short stretches of nucleic acid [37,38,39,40,41], and due to the presumed upregulation of dNTP synthesis indicated by drug-dependent cytoplasmic localization of Rnr4-GFP (Figure 3a–c), we hypothesized that KP1019 would display synergy with the chemotherapeutic agent 5-fluorouracil (5-FU), which inhibits synthesis of the nucleotide thymidylate [42]. Given the ability of KP1019 to bind nitrogenous bases of nucleotides and nucleic acids in vitro [43,44], we minimized direct chemical reactions between KP1019 and the nucleobase analog 5-FU by treating cells with the drugs sequentially, as described in the Materials and Methods section. Figure 3f,g present a heat map of growth inhibition, as well as the interaction landscape between KP1019 and 5-FU, as determined by the zero interaction potency (ZIP) model [45]. We observed a pronounced peak in the interaction landscape between 30–300 µg/mL KP1019 and 0.3–3 µg/mL 5-FU (Figure 3g). Within this 3 × 3 dose window of maximal synergy, the average delta (δ) score, a measure of the observed interaction between drugs relative to the expected value predicted by the ZIP model [45], was 14 ± 2 (*n* = 3). This region included an even higher localized peak, consistent with significant synergy across a roughly 10-fold range of concentrations for each drug.

### 2.3. pH-Dependence of KP1019 Bioactivity

In addition to DNA, the screen implicated vacuolar acidification as a modulator of KP1019 tolerance (Figure 2g,j). In yeast, the V-type ATPase (V-ATPase) resides in the vacuolar membrane, which is the yeast equivalent of the lysosome [46]. Given that loss of V-ATPase function can lower cytosolic pH in yeast [47,48,49] and that previous chemical analyses showed that low pH increases KP1019 stability and nucleotide binding in simple buffer systems [43,50], the findings from the screen prompted us to more broadly examine the impact of pH modulation on KP1019 tolerance. Specifically, we tested the effects of altered pH on drug toxicity. Consistent with prior chemical analyses of KP1019 [51], we used a citrate buffer to control the pH of the yeast growth medium. Importantly, citrate can buffer across a wide range of pH levels, including those low enough to maintain a sufficient proton gradient to support nutrient transport across the yeast plasma membrane and to avoid rapid precipitation of the drug [52,53].

**Figure 4:**
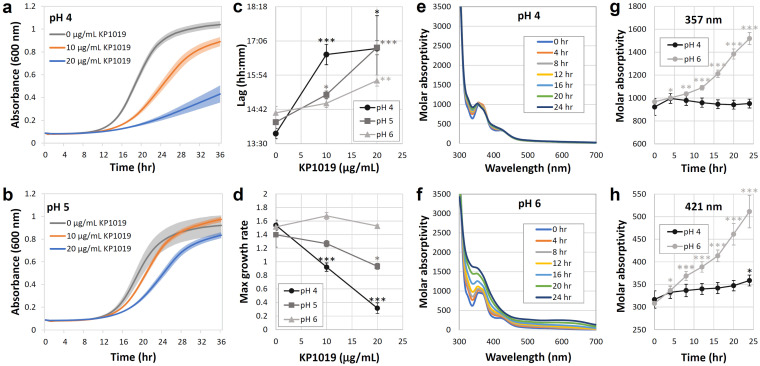
pH modulates KP1019 toxicity. (**a**,**b**) Growth of wild-type yeast was measured over time in the presence of varying concentrations of KP1019 in pH 4 (**a**) and pH 5 (**b**) media buffered with 50 mM citrate. Lines indicate average absorbance at 600 nm, and shading surrounding lines corresponds to the standard error of five biological replicates. (**c**,**d**) The (**c**) lag and (**d**) maximum growth rates (A_600_ × 1000/min, where A_600_ refers to absorbance at 600 nm) from panels (**a**,**b**) and an analogous trials at pH 6 were plotted as a function of drug concentration. Graphs depict the mean and standard error of five biological replicates. Multiple linear regression analysis revealed pH and KP1019 concentration had strong collective effects on lag (F(2, 42) = 18.59, *p* < 0.001, R^2^ = 0.47, R^2^_adj_ = 0.44) and maximal growth rate (F(2, 42) = 33.64, *p* < 0.001, R^2^ = 0.62, R^2^_adj_ = 0.62). * *p* < 0.05, *** *p* < 0.001 relative to no drug control for the same pH as determined by Student’s *t*-test. (**e**–**h**) UV–vis spectrophotometry assessed the stability of 0.25 mM KP1019 in citrate-buffered yeast media. (**e**,**f**) UV–vis spectra at 4 h intervals at pH 4 (**e**) and 6 (**f**). Spectral graphs depict one representative trial per condition. (**g**,**h**) Molar absorptivity at KP1019 LMCTs is graphed as a function of time. Data correspond to the mean and standard deviation of three trials. Multiple linear regression analysis revealed pH and time had strong collective effects on absorbance at 357nm (F(2, 39) = 32.92, *p* < 0.001, R^2^ = 0.63, R^2^_adj_ = 0.61) and at 421nm (F(2, 39) = 48.27, *p* < 0.001, R^2^ = 0.71, R^2^_adj_ = 0.7). * *p* < 0.05, ** *p* < 0.01, *** *p* < 0.001 relative to time 0 for the same pH as determined by Student’s *t*-test.

As seen in Figure 4a–d, we analyzed the effects of KP1019 on the growth curves of wild-type yeast in citrate-buffered media at pH 4, 5, and 6. The drug exhibited the strongest growth inhibition at pH 4, which is slightly lower than the pH of unbuffered synthetic defined media. In these liquid assays, KP1019 concentration was a significant (*p* < 0.001) predictor of both lag (time to maximum slope) (Figure 4c) and maximum growth rate (Figure 4d). Although the impact of 10 µg/mL KP1019 on lag was blocked at pH 6, multiple linear regression analysis revealed that the overall impact of pH on lag only approached significance (*p* = 0.07). In contrast, pH was a significant predictor (*p* < 0.001) in the model for maximum growth rate. These data are consistent with the model that modulators of drug activity can impact CPPs to different extents.

The higher bioactivity of KP1019 at low pH correlates with greater drug stability as assessed spectrophotometrically. As seen in Figure 4e,f, the UV–visible spectrum for KP1019 in buffered yeast media initially includes characteristic peaks at 357 and 421 nm, which result from ligand to metal charge transfer (LMCT) [54]. In media buffered to pH 4, KP1019 remains soluble, and its UV–visible spectrum undergoes only modest changes during the timeframe of the experiment (Figure 4e,g,h). In contrast, at pH 6, the LMCT peaks are rapidly lost, becoming less distinct as water replaces the chloride ligands of the drug and molar absorptivity increases [46]. These rapid changes suggest ligand exchange with components in the buffered growth medium (Figure 4f–h). Moreover, the lack of an isosbestic point and the increasing absorbance at high wavelengths are consistent with the formation of multiple reaction intermediates and ruthenium-containing precipitates, respectively. Thus, the stability of the drug in solution correlates with its toxicity.

### 2.4. KP1019 Induces Ribosome Biogenesis Stress

**Figure 5:**
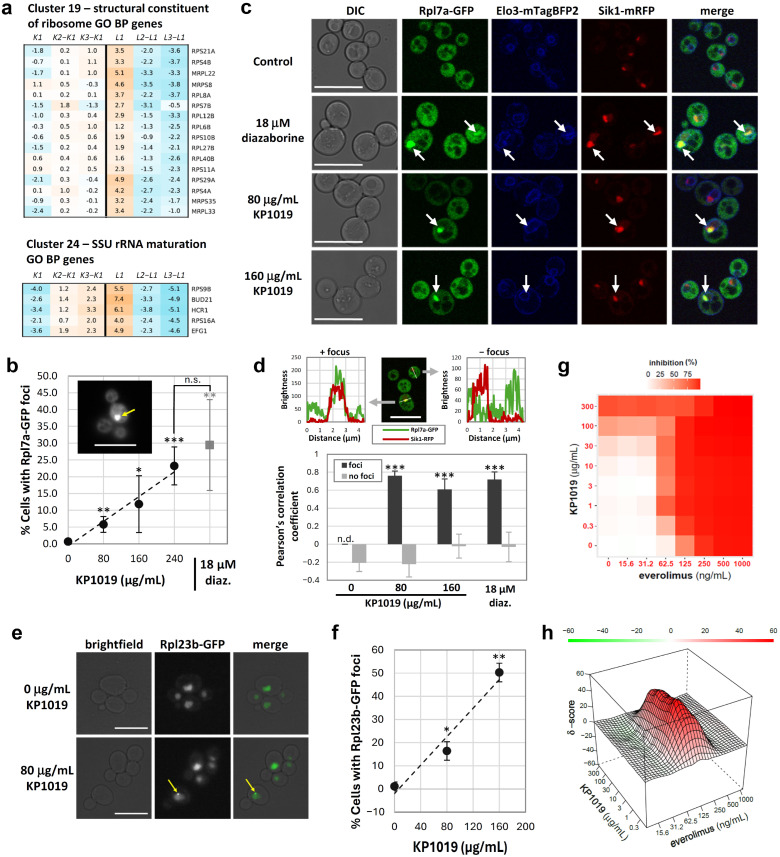
KP1019 perturbs ribosomal biogenesis. (**a**) Ribosome-related gene deletions suppressed KP1019 activity. Heat maps include all genes associated with the indicated GO terms. Other genes from each cluster were omitted for brevity. Full cluster details are available in Supplemental Files S4 and S5. (**b**) Exponentially growing yeast expressing Rpl7a-GFP were treated with KP1019 or diazaborine for 3 h prior to fluorescence microscopy. Yellow arrow indicates an Rpl7a-GFP focus. Rpl7a-GFP localization was scored for 100–600 cells per trial per condition. Data presented are the mean and standard deviation for four trials. Linear regression analysis revealed a strong relationship between drug concentration and Rpl7a-GFP focus formation (R^2^ = 0.74, F(1, 14) = 40.85, *p* < 0.001). Scale bar, 10 μm. * *p* < 0.05, ** *p* < 0.01, *** *p* < 0.001 relative to negative control, and ns means not significant, as determined by Student’s *t*-test. (**c**) Exponentially growing yeast cells expressing Rpl7a-GFP, Elo3-mTagBFP2, and Sik1-mRFP were treated with KP1019 or diazaborine for 3 h prior to live cell confocal microscopy. Arrows indicate Rpl7a-GFP foci. Scale bars, 10 μm. (**d**) GFP and RFP co-localization was assessed using ImageJ’s Plot Profile tool to determine brightness along a line extending across the cell and through the nucleolus (Sik1-RFP signal). Scale bar, 10 μm. Bars represent average Pearson’s correlation coefficient ± 2× standard error; *n* = 11–14 per cell type (foci vs. no foci) per condition. *** *p* < 0.001 relative to cells without foci for the same condition, as determined by Student’s *t*-test. (**e**) Exponentially growing Rpl23b-GFP yeast cells were treated with KP1019 for 3 h prior to fluorescence microscopy. Yellow arrow indicates an Rpl23b-GFP focus. Scale bars, 10 μm. (**f**) Rpl23b-GFP focus formation was scored for 120–190 cells per trial per condition. Data presented are the mean and standard deviation for three trials. Linear regression analysis revealed a strong relationship between drug concentration and Rpl23b-GFP focus formation (R^2^ = 0.94, F(1, 7) = 104.42, *p* < 0.001). * *p* < 0.05, ** *p* < 0.01 relative to negative control, as determined by paired *t*-test. (**g**,**h**) Exponentially growing wild-type yeast cells were treated with varying concentrations of KP1019 for 3 h prior to 18–24 h of growth in the presence of everolimus. The response matrix (**g**) and interaction landscape (**h**) represent the average of three biological replicates. Results of individual trials are available in Supplemental File S2 (Figure S5).

As shown in Figure 2j, GO term analysis of KP1019-suppressing gene deletions revealed ribi and translation as modulators of drug activity. Excerpts from two representative clusters are shown in Figure 5a. Adjusted *L* z-scores for these clusters show that these strains are resistant to KP1019, and thus deletion of the corresponding genes suppresses the growth inhibitory activity of KP1019.

Interestingly, a previous study uncovered a positive correlation between the expression of RP genes and sensitivity to platinum-based chemotherapeutics that cause ribi stress [55]. To determine whether suppression of KP1019 activity in yeast lacking certain RP or ribi genes was indicative of KP1019-dependent induction of ribi stress, we measured KP1019-dependent changes in the subcellular localization of Rpl7a-GFP. In unstressed cells, Rpl7a-GFP is cytoplasmic, but induction of ribi stress causes it to accumulate in the nucleoplasm, nucleolus, or both [56,57]. As seen in Figure 5b, the percentage of cells with Rpl7a-GFP foci increases as the concentration of KP1019 increases, reaching levels similar to those for the well-established ribi stress agent diazaborine [57]. To determine whether Rpl7a-GFP foci formed following KP1019 treatment correspond to the nucleolus, nucleoplasm, or an alternate structure, we conducted confocal live cell imaging with yeast expressing Rpl7a-GFP; mRFP-tagged Sik1, an evolutionarily conserved nucleolar protein [58,59,60]; and mTagBFP2-tagged Elo3, a fatty acid elongase found in endoplasmic reticulum (ER) membranes [61,62]. In yeast, ER membranes are contiguous with the nuclear envelope and thus surround the yeast nucleus, and the cortical ER lies just beneath the yeast plasma membrane [62]. As seen in Figure 5c,d, the Rpl7a-GFP foci did not fill the entire nucleus but instead overlapped with the nucleolar marker Sik1-mRFP [60], suggesting KP1019 impacts an early stage in ribi.

To further support the model that KP1019 induces ribi stress, we examined the impact of the drug on the localization of Rpl23b-GFP. Importantly, Rpl23b is a component of the intranuclear quality control compartment (INQ), and activation of the ribosome assembly stress response can cause Rpl23b-GFP to form foci [63]. Here, we show that KP1019 induces Rpl23b-GFP focus formation (Figure 5e,f), supporting the model that the drug impacts ribi.

Given this new evidence that KP1019 may interfere with ribi, we examined whether KP1019 is synergistic with pharmacological TOR pathway inhibition, which is thought not to cause ribi stress but does impede translation through other means, including reduced transcription of RP genes and altered translation initiation [64,65]. Importantly, previous studies showed that perturbation of ribosomes with drugs acting at different levels can result in substantial synergy [66,67]. To be consistent with the KP1019-5-FU synergy assay (Figure 3f,g), yeast cells were treated with KP1019 and everolimus sequentially, as described in the Materials and Methods section. As shown by the growth inhibition heat map and drug interaction landscape (Figure 5g,h), there is a ridge of potent synergy between a wide range of KP1019 concentrations and 62.5–125 ng/mL of the TOR inhibitor everolimus. The summary synergy score for the entire landscape was 8.9 ± 2.1 (*n* = 3), with the δ score remaining over 40 along much of the ridge crest, supporting a model wherein even KP1019 concentrations too low to alter Rpl7a-GFP localization sufficiently perturb ribi to potentiate the effects of everolimus in yeast.

## 3. Discussion

Here, we present the results of an unbiased quantitative phenomic screen of over 4000 yeast strains, each lacking a different non-essential gene. Expected biological impacts of KP1019 were recapitulated, as loss of DNA damage repair pathways enhanced the drug’s effects (Figure 2 and Figure 3), which is consistent with previous studies of cancer and yeast [9,14,15,16,17,68]. In addition to implicating DNA damage, vacuolar acidification, and ribi as modulators of KP1019 activity, the screen identified numerous KP1019-enhancing and -suppressing gene deletions that were not associated with enriched GO terms. For example, strains lacking the mitochondrial magnesium homeostasis genes *MRS2* and *MME1* [69,70] were identified as KP1019-resistant. Given previous reports that KP1019 accumulates in the mitochondria of yeast and ovarian cancer cells [18,20], this finding is intriguing. It suggests that future studies could leverage the results of this screen to gain additional insight into other mechanisms influencing KP1019 activity.

### 3.1. Verification of DNA as One of the Targets of KP1019

Despite being carried out at lower concentrations of KP1019 than previous high-throughput yeast studies [15,17,26], this work verified that DNA is a major target of the drug in yeast (Figure 2 and Figure 3). This result is consistent with the ability of KP1019 to bind nucleotides in vitro [43], bind DNA in ovarian cancer cells [18], and cause DNA strand breaks in colorectal cancer cells [9]. The utility of yeast as a model organism for characterizing KP1019 is also supported by the observed synergy between KP1019 and 5-FU (Figure 3g), which impairs DNA replication and repair by inhibiting thymidylate synthase [42]. This finding is analogous to the synergy seen between 5-FU and BOLD-100, the sodium salt analog of KP1019, in Lovo and HCT-116 colon cancer cell lines [71]. More recently, a large-scale screen of cancer cell lines compared the drug sensitivity profile of BOLD-100 to that of screened drugs available in the Genomics of Drug Sensitivity in Cancer (GDSC) database [72]. When examining data from solid tumor cell lines, Park et al. noted that the BOLD-100 response profile was significantly correlated with 12 drugs (false discovery rate < 0.01) [72]. This list includes bleomycin, 5-FU, and doxorubicin, which target DNA either directly or indirectly. Likewise, analysis of the response profiles of liquid tumor cell lines revealed significant correlations between BOLD-100 and the DNA-cross-linking agent carmustine, as well as pyridostatin, which damages DNA by disrupting replication and transcription [73]. These results support the conclusion that DNA is a target of the *trans*-[tetrachlorobis (1*H*-indazole)ruthenate(III)] anionic complex regardless of the counterion.

Importantly, the present study extends existing models for KP1019-induced DNA damage by implicating Pso2 in the repair process (Figure 3d,e). Since Pso2 is central to ICL repair in yeast [36,37], this result is consistent with studies showing that KP1019 induces ICLs in biophysical DNA-binding assays, as well as in ovarian cancer cells [68,74]. Moreover, the correlation between cancer cell line responses to the KP1019 analog BOLD-100 and the DNA cross-linking agent carmustine further implicates ICL formation as a physiologically relevant drug impact [72]. Since tolerance and repair of ICLs can involve HR and TLS [36,37,75], the findings we present here align well with current and prior work showing that yeast strains lacking these repair pathways are hypersensitive to KP1019 (Supplemental File S5) [14]. Since HR involves formation of double-strand breaks in DNA, recombination-mediated repair of ICLs may also explain some of the KP1019-dependent DNA fragmentation observed in single-cell gel electrophoresis assays of colorectal cancer cells [9].

Ultimately, the role for ICL repair in KP1019 tolerance may have significant clinical implications, as yeast Pso2 has five human orthologs, two of which—*DCRLE1A*/*SNM1A* and *DCRLE1B*/*SNM1B*—have roles in ICL repair [36]. Notably, knockdown of *DCLRE1B* expression increases cisplatin sensitivity in ovarian cancer cells in vitro [76], and data-mining and bioinformatic analyses have shown that *DCLRE1A* is part of a seven-gene module, low expression of which is associated with better clinical outcomes for ovarian cancer patients [77]. Although additional studies are needed, expression of *DCLRE1A*, *DCLRE1B*, or both may be able to serve as a prognostic factor for patient responses to KP1019 and similar metallodrugs.

### 3.2. pH Homeostasis as a Physiologically Relevant Modulator of KP1019 Toxicity

In addition to verifying DNA as one of the targets of KP1019, the screen uncovered vacuolar acidification as a modulator of sensitivity to the drug. Although additional studies are needed to determine the mechanism or mechanisms at play, the observed KP1019 sensitivity may be related to the broad metal sensitivity of yeast lacking V-ATPase function. For example, previous studies have shown that loss of V-ATPase function in yeast increases sensitivity to diverse metal-containing compounds, including ZnSO_4_, CrO_3_, K_2_CrO_4_, Pb(NO_3_)_2_, AlCl_3_, and cisplatin [78,79,80,81,82]. Previous studies have also shown that deletion of genes encoding subunits of the V-ATPase lowers cytosolic pH and increases intracellular reactive oxygen species, which sensitizes these mutants to both organic acid and oxidative stress [47,48,49,83,84,85,86]. In the phenomic screen presented here, KP1019-enhancing deletions were not enriched for GO terms related to oxidative stress. Thus, as a preliminary follow-up to the screen, we focused on pH as a modulator of KP1019 bioactivity.

Analogous to the correlation between low cytosolic pH and enhanced KP1019 sensitivity of V-ATPase mutants, we observed that low extracellular pH correlated with greater drug-dependent growth inhibition in wild-type yeast (Figure 4a–d). This variation in KP1019 bioactivity likely relates to its increased stability at low pH. Although the pH-dependence of KP1019 stability in simple buffer systems is well established [50,87], here, we demonstrate similar trends in more complex yeast media (Figure 4e–h). Although cultured human cells appear unable to withstand the extracellular pH range of 4–6 that we tested with yeast [88,89,90], when KP1019 is pre-incubated with Dulbecco’s Modified Eagle Medium cell culture medium, which is buffered to pH ~7.4, the drug is unstable, and drug uptake is reduced by over 90% in HepG2 cells [21]. The decreased stability we observed in yeast media at pH 5 and 6 may have caused analogous decreases in drug uptake.

Interestingly, there is a modest but reproducible positive correlation between extracellular and cytosolic pH in yeast [91,92,93]. So, the greater toxicity of KP1019 in low pH media may be influenced by both extracellular and intracellular effects. In fact, the KP1019 sensitivity of yeast with low cytosolic pH due to either lack of V-ATPase function or growth at low pH may reflect pH-dependent increases in KP1019 binding to intracellular targets since the drug binds more readily to GMP in biophysical assays run at lower pH [43,87]. This model is particularly attractive given the low extracellular pH of many solid tumor microenvironments and the corresponding decrease in cytosolic pH that has been observed in the hypoxic and acidic cores of cancer organoids in vitro [94,95,96]. If future in vitro studies of cancer cells demonstrate that low cytosolic pH does in fact increase KP1019 efficacy, subsequent preclinical research might explore whether KP1019 bioactivity in cancer organoids with acidic cores can be increased through co-administration with either the pH modulating drug amiloride, which inhibits Na^+^-H^+^ exchangers, or the proton ionophore nigericin, both of which have their own anticancer properties [97,98,99].

### 3.3. Perturbation of Ribosomal Biogenesis by KP1019

An unanticipated finding of this study is that high concentrations of KP1019 appear to induce ribi stress in yeast (Figure 5). Although KP1019-dependent changes in RP localization occurred at concentrations substantially higher than those used in the screen (80 vs. 10–20 µg/mL), the screen intentionally relied on low concentrations so that growth parameters for the majority of deletion strains could be quantified. Moreover, the concentrations tested in ribi stress microscopy assays are in line with other studies. For example, we observed statistically significant KP1019-dependent changes in Rpl7a-GFP and Rpl23b-GFP localization beginning at 80 µg/mL (134 µM), whereas published transcriptomic and proteomic studies have documented KP1019-dependent increases in RP and ribi protein expression in yeast at 50 and 80 µg/mL, respectively [17,26]. Moreover, a screening of 319 cancer cell lines determined that the pan-cancer median IC_50_ for BOLD-100 was 149 µM [72], which is higher than the concentration of KP1019 required to significantly alter RP localization in yeast. Likewise, a transcriptomic study of 100 µM BOLD-100 revealed drug-dependent increases in expression of RP genes [100]. Thus, the KP1019 concentrations used in the microscopy-based assays for ribi stress in yeast likely fall within physiologically relevant ranges.

Importantly, this finding has the potential to reconcile many prior observations regarding KP1019 and BOLD-100 (Figure 1). For example, a recent transcriptomic study of yeast showed that perturbing ribosome formation by repressing RP uL4 increases the expression of other RP genes [101]. This result is consistent with a model wherein drug-induced ribi stress may be responsible for the KP1019- and BOLD-100-dependent upregulation of RP genes in yeast [17,26,100]. Importantly, the interaction between translation machinery expression and drug response does not appear to be cell line-specific, as a screening of over 300 cancer cell lines uncovered a positive correlation between expression of ribosome-related genes and sensitivity to BOLD-100 [72]. This result parallels that of a previous study showing that elevated levels of translation machinery are associated with sensitivity to platinum-based chemotherapeutics that perturb ribi [55].

The observed synergy with the TOR pathway inhibitor everolimus is consistent with the conclusion that KP1019 induces physiologically relevant ribi stress (Figure 5g,h), as combination therapies that perturb ribosome synthesis at different levels have previously been shown to result in substantial synergy [66,67]. Moreover, our results are in line with a prior report of synergy between everolimus and BOLD-100 in the MKL-1 Merkel cell carcinoma cell line [71]. Interestingly, a previous study noted only additive effects between KP1019 and rapamycin in HeLa cells and reported that the interaction between rapamycin and KP1019 varied by drug ratio in a yeast-based assay [26]. The differences between the findings we present here and those of Golla et al. [26] could be due to a variety of factors, including differences in the pharmacokinetics of rapamycin and everolimus [102]. Alternatively, the conflicting results may be explained by variations in experimental design, including the order in which drugs were administered, our reliance on strictly quantitative approaches, and our testing of a wider range of drug concentrations to more finely map the KP1019-everolimus interaction landscape.

Given the precipitous sensitivity of deletion strains with defects in DNA repair and the DNA damage response, and given that the concentration of KP1019 required to induce changes in RP localization was substantially higher than those used in the screen, we propose that ribi is a secondary target of KP1019. Future research can apply alternate approaches to verify that KP1019 causes ribi stress and to explore whether this stress is a result of insults to rDNA transcription, rRNA processing, or subunit assembly. Given that KP1019 damages DNA, disruption of rDNA transcription seems especially plausible because dysregulation of the ratio of rRNA and RPs in yeast has been shown to activate the transcription factor Hsf1 [103,104], as has KP1019 treatment [17]. Currently, it is also unclear whether the translation-related stress caused by KP1019 is exacerbated by interactions between the drug and tRNA, which have been observed in vitro [105].

An especially intriguing question is why there is a positive correlation between RP abundance and sensitivity to metal-based drugs that induce ribi stress [55,72]. Here, we observed that loss of certain RPs and ribi factors results in KP1019 resistance. This form of ribosome deficiency often leads to a slower intrinsic proliferation rate [106], which may reflect ribi perturbations, such as those observed upon loss of Rps29 [107]. Thus, these KP1019-resistant deletion strains may be genetically pre-conditioned to withstand the impacts of the drug on ribi. It has also been proposed that high levels of translation machinery may reflect a cell line’s “addiction” to protein synthesis, which, in turn, increases sensitivity to ribi perturbation [55]. If this is the case, then deletion of certain RP genes may break the translation addiction, which likely exists in rapidly dividing wild-type yeast. Alternatively, loss of non-essential RPs may alter the pool of mRNAs being translated (i.e., the translatome). For example, loss of an RP paralog or homogenization of a pair of RP paralogs in yeast has been shown to alter the translation index of subsets of genes that likely explain the strains’ altered resistance to certain drugs and osmotic stress [108]. Importantly, translation reprogramming caused by ribosome heterogeneity is not limited to yeast, and its relevance to cancer initiation, progression, and relapse is an important area of ongoing research [109,110].

## 4. Materials and Methods

### 4.1. Yeast Strains and Plasmids

The yeast deletion strain library was obtained from Research Genetics (Huntsville, AL, USA) and is in the BY4741 genetic background, which is described in Table 3. Additional strains used in this study are also listed in Table 3. The Rpl7a-GFP Elo3-mTagBFP2 strain was constructed using standard lithium acetate transformation to incorporate integrating DNA fragments and a plasmid into the Rpl7a-GFP strain [111]. Specifically, *TRP1* was first disrupted with *kanMX* using the previously described marker swap cassette [112]. Elo3 was tagged with mTagBFP2 using the previously described construct obtained from AddGene (Watertown, MA, USA) [62]. This strain was then transformed with the previously described *SIK1-mRFP* plasmid [60].

### 4.2. Yeast Media and Drugs

Yeast strains were maintained on the rich medium YPD (1% yeast extract, 2% bacto-peptone, 2% dextrose) (BD Difco, Franklin Lakes, NJ, USA). The night before and during experiments, yeast strains were grown in the synthetic dextrose complete (SDC)-defined medium (0.67% bacto yeast nitrogen base w/o amino acids (BD Difco), 2% dextrose (Fisher Scientific, Waltham, MA, USA)). A complete mixture of amino acids and nitrogenous bases (Sigma-Aldrich, St. Louis, MO, USA) was supplemented as described previously [115]. When appropriate, 20 g/L agar (BD Difco) was added to solidify the media. During strain construction, the media were supplemented with G418 (Teknova, Hollister, CA, USA), or an amino acid was dropped out to allow selection of transformants. Specifically, tryptophan was omitted from the growth medium when selecting for genomic integration of the *ELO3-mTagBFP2* construct, and methionine was omitted when selecting for transformation with the *SIK1-mRFP* plasmid.

KP1019 was synthesized as described previously [14,17]. 5-FU and everolimus were purchased from Sigma-Aldrich. Diazaborine was from Calbiochem (San Diego, CA, USA). For all experiments, KP1019 was dissolved directly in yeast media to avoid the previously reported interference of DMSO with its stability and bioactivity [116].

### 4.3. Phenomic Screening, Clustering, and Gene Ontology Enrichment Analysis

The yeast deletion collection was screened via the quantitative high-throughput cell array phenotyping (Q-HTCP) method [4,6,117,118]. Briefly, a Sciclone 3000 liquid handling robot from Caliper Life Sciences (Hopkinton, MA, USA) was used to transfer the arrayed deletion collection onto solidified SDC containing 0, 10, and 20 µg/mL KP1019. An Expression 10,000 XL scanner from Epson (Los Alamitos, CA, USA) was used to collect transmitted light images every 3–4 h until the carrying capacity (*K*) was reached, which was approximately 5 days. MATLAB version 2011a (MathWorks, Natick, MA, USA) was used for image analysis; the time stamp intervals between image acquisition were plotted vs. average pixel intensity for each spot culture; and the resulting data for each growth curve was fit to a logistic growth equation *G(t) = K/(1 + e ^−r(t−L)^)* to obtain the parameter values for *K*, the maximum specific growth rate (*r*), and the time (t) at which *K*/2 is reached (*L*). The cell proliferation parameters obtained are available in Supplemental File S3.

After filtering out problematic images involving cell arrays that appeared to have experimental artifacts, such as patterns of poor-quality spot cultures (Supplemental File S1), data analysis was conducted in WinPython version 2025-3. For all high-quality data, the fitness effect of the gene deletion at each drug concentration (0, 10, and 20 ug/mL KP1019) was standardized as a z-score using the formula *Z-score_mut_cpp = (CPP_mut − CPP_deletion array_median)/stdev (384reference_array)*, where “*CPP_mut*” refers to the CPP for a specific deletion at a specific KP1019 concentration, “*CPP_deletion_array_median*” refers to the median CPP for all deletion strains on the same 384-strain array, and “*stddev (384reference_array)*” refers to the standard deviation from a complete array of 384 reference strain cultures.

To assess whether the fitness effect of the gene deletion was specific to the response to KP1019 perturbation, the z-score in the absence of the drug was subtracted from the z-score in the presence of the drug. The resulting adjusted z-scores reflect the gene–drug interaction. KP1019-suppressing interactions were defined as having positive adjusted *K* z-scores, negative adjusted *L* z-scores, or both. KP1019-enhancing interactions were defined as having negative adjusted *K* z-scores, positive adjusted *L* z-scores, or both. The adjusted z-scores were analyzed by hierarchical clustering, and the Bayesian information criterion was used to split the hierarchical cluster tree into an optimal number of clusters, determined to be 31. GO term overrepresentation for each cluster was analyzed by Fisher’s exact test via the GOATOOLS Python library version 1.5.2 [119]. The Benjamini–Hochberg (BH) procedure was used to correct for false discovery. The background gene set for the GO term analysis comprised the 4577 unique gene deletions for which high-quality data was obtained (list is available in Supplemental File S3). Within the deletion collection, some open reading frame (ORF) deletion strains are present multiple times. These deletions were handled as a single entry in the background gene set used for GO term analysis. Duplicate gene deletions that fell outside of clusters were ignored. If duplicate gene deletion strains were present in multiple clusters, each entry was used in the GO enrichment calculation for its respective cluster.

### 4.4. Microscopy

For microscopy experiments, yeast cells were cultured overnight in SDC and then sub-cultured to mid-log phase (absorbance at 600 nm (A_600_) 0.3–0.8) in SDC. Yeast cultures were then diluted back to the early- to mid-log phase (A_600_ 0.2–0.5), and the indicated concentrations of KP1019 or diazaborine were added. Cells were incubated with KP1019 or diazaborine for 3 h prior to visualization by microscopy.

Prior to visualizing Rnr4-GFP and scoring budding index, clumps of cells were disrupted by sonication for approximately 10 s at 4% intensity output (Sonicator Dismembrator Model 100, Fisher Scientific). Images were captured using a 20× objective with the LSM710 Confocal Imaging System with ZEN software version 2012 SP1 HF9 (Carl Zeiss AG, Oberkochen, Germany). Cellular morphology was scored based on bud presence and size, as described previously [15]. Specifically, large buds were approximately two-thirds the size of the mother cell, small buds were approximately one-third the size of the mother cell, and medium buds were in between. Rnr4-GFP localization was scored as nuclear or cytoplasmic. At least 250 cells were counted per sample for each of three biological replicates.

Initial localization of Rpl7a-GFP and Rpl23b-GFP was visualized using the EVOS FL and EVOS M5000 imaging systems (Advanced Microscopy Group, Mill Creek, WA, USA), outfitted with a 100× objective lens and a GFP light cube. The percentage of cells with GFP foci was scored. At least 100 cells were counted per condition per trial. Rpl7a-GFP localization was refined using the Rpl7a-GFP Elo3-mTagBFP2 strain transformed with the Sik1-mRFP plasmid. Images were captured using the oil immersion 63× objective lens of a Leica Microsystems TCS SP8 Spectral Confocal Microscope and the Leica LAS X control software version 3.5.7 (Leica Microsystems, Wetzlar, Germany). Localization patterns under each condition were verified with at least three biological replicates. Validation of Rpl7a-GFP and Sik1-RFP overlap within foci was carried out in ImageJ version 1.54r [120] by first creating linear regions of interest (ROIs) that each spanned a cell while passing through the nucleolus (Sik1-RFP signal). The ImageJ Plot Profiler tool was then used to gather data on the brightness of each fluorophore as a function of position along the ROI. Pearson’s correlation coefficient for each ROI was calculated in Excel.

### 4.5. Drug Sensitivity and Synergy Assays

The IC_50_ of KP1019 was determined as described previously [14]. Briefly, KP1019 was dissolved in SDC yeast media and then subjected to a two-fold serial dilution. Overnight *Saccharomyces cerevisiae* cultures were normalized to A_600_ = 0.1 and then diluted 20-fold more in SDC. An equal volume of cell suspension was added to the serially diluted KP1019. Plates were incubated at 30 °C for 18–24 h before end-point absorbance (630 nm) was measured with a BioTek ELX800 microplate reader (BioTek, Winooski, VT, USA). The drug concentration that inhibited growth by 50% was determined by fitting the data with a four-parameter logistic curve in SigmaPlot (Grafiti, Palo Alto, CA, USA).

Growth curve assays were conducted as described previously [116]. Key differences with the IC_50_ assay include the use of mid-log phase cultures to reduce the duration of the lag phase and the prevention of condensation with pre-treatment of microtiter plate lids [121]. A BioTek Synergy LX plate reader controlled by Biotek Gen5 software version 3.10.06 (Agilent Technologies, Santa Clara, CA, USA) held the plates at room temperature and measured absorbance at 600 nm every 30 min for 36 h. Lag times (the time to maximum slope) and maximum growth rates were determined by Biotek Gen5 software version 3.10.06 (Agilent Technologies).

Synergy assays were conducted as described previously [17]. Briefly, mid-log phase cultures of wild-type yeast (BY4742) were treated with the indicated concentrations of KP1019 for 3 h. After washing with SDC, normalizing A_600_, and diluting 10-fold, yeast samples were incubated with the indicated concentrations of 5-FU or everolimus for 18–24 h at 30 °C. Absorbance at 600 nm was then measured with a BioTek Epoch 2 plate reader controlled by BioTek Gen6 software version 1.03.01 (Agilent Technologies). The resulting data were analyzed using SynergyFinder 3.0 [122].

### 4.6. UV–Vis Spectrophotometry

Absorbance spectra were studied as described previously [116]. Briefly, 0.25 mM of KP1019 was dissolved directly in SDC buffered to the indicated pH by the addition of 50 mM of citrate buffer. Data was collected at 30 °C every hour for 24 h using a BioTek Epoch 2 plate reader (Agilent Technologies). Absorbance was converted to molar absorptivity by dividing by the concentration of KP1019. For clarity, graphs include scans collected at 4 h intervals.

## Figures and Tables

**Table 1:** Ten most KP1019-enhancing gene deletions.

Deleted Gene/Open Reading Frame	Description	Adjusted z-Score (*K2* − *K1*)
*RAD9*	DNA damage-dependent checkpoint protein	−10.4
*XRS2*	Component of the Mre11 complex, which is involved in double-strand break repair	−9.3
*YMR001C-A*	protein of unknown function	−9.3
*RAD5*	DNA helicase/Ubiquitin ligase; involved in error-free branch of DNA damage tolerance (DDT) pathway	−9.2
*RAD55*	Protein involved in the recombinational repair of double-strand breaks in DNA	−8.6
*MRE11*	Nuclease subunit of the MRX complex that functions in repair of DNA double-strand breaks	−8.6
*REV7*	Accessory subunit of DNA pol zeta; involved in translesion synthesis during post-replication repair	−8.6
*RAD51*	Strand exchange protein involved in the recombinational repair of double-strand breaks in DNA	−8.4
*RAD57*	Protein involved in the recombinational repair of double-strand breaks in DNA	−8.0
*YLR235C*	Dubious open reading frame; partially overlaps with DNA topoisomerase III gene *TOP3*	−7.8

Deletion strains were selected for inclusion in this table due to having one of the 10 lowest adjusted *K* z-scores at 10 µg/mL. Descriptions are often verbatim excerpts from the Yeast Genome Database [29].

**Table 2:** Ten most KP1019-suppressing gene deletions.

Deleted Gene/Open Reading Frame	Description	Adjusted z-Score (*L3* − *L1*)
*RPL36B*	Ribosomal 60S subunit protein L36B	−10.0
*SOM1*	Subunit of the mitochondrial inner membrane peptidase (IMP) required for maturation of mitochondrial proteins of the intermembrane space	−9.9
*YMR242W-A*	Putative protein of unknown function	−9.5
*RPL39*	Ribosomal 60S subunit protein L39	−9.2
*YKL169C*	Dubious open reading frame; partially overlaps with mitochondrial ribosomal protein gene MRPL38	−9.1
*CHO2*	Phosphatidylethanolamine methyltransferase (PEMT)	−8.9
*CCR4*	Component of the CCR4-NOT complex, which is involved in regulation of gene expression	−8.8
*YGR160W*	Dubious open reading frame, overlaps with nucleolar protein gene NSR1, which is required for pre-rRNA processing and ribosomal biogenesis	−8.4
*AAH1*	Adenine deaminase (adenine aminohydrolase); converts adenine to hypoxanthine	−8.2
*RPL37A*	Ribosomal 60S subunit protein L37A	−8.1

Deletion strains were selected for inclusion in this table due to having one of the 10 lowest adjusted *L* z-scores at 20 µg/mL. Descriptions are often verbatim excerpts from the Yeast Genome Database [29].

**Table 3:** Yeast strains used in this study.

Strain	Genotype	Refs.
BY4741	*MAT***a** *his3Δ1 leu2Δ0 met17Δ0 ura3Δ0*	[113]
BY4742	*MAT***α** *his3Δ1 leu2Δ0 lys2Δ0 ura3Δ0*	[113]
*pso2* *Δ*	*pso2::kanMX* in BY4742	[113]
Rnr4-GFP	*RNR4-GFP::HIS3* in BY4741	[114]
Rpl7a-GFP	*RPL7A-GFP::HIS3* in BY4741	[114]
Rpl7a-GFP Elo3-mTagBFP2	*RPL7A-GFP::HIS3 trp1::kanMX ELO3-mTagBFP2::TRP1* in BY4741	This study
Rpl23b-GFP	*RPL23B-GFP::HIS3* in BY4741	[114]

## Data Availability

The original contributions presented in this study are included in the article/Supplementary Material. Further inquiries can be directed to the corresponding author.

## Supplementary Materials

The following supporting information can be downloaded at https://www.mdpi.com/article/10.3390/ijms27073275/s1.

## Abbreviations

The following abbreviations are used in this manuscript:

BOLD-100	Sodium *trans*-[tetrachlorobis(1*H*-indazole)ruthenate(III)]
CPP	Cell proliferation parameter
5-FU	5-fluorouracil
GO	Gene ontology
HR	Homologous recombination
IC_50_	Half-maximal inhibitory concentration
ICL	Interstrand cross-link
KP1019	Indazolium *trans*-[tetrachlorobis(1*H*-indazole)ruthenate(III)]
LMCT	Ligand to metal charge transfer
Ribi	Ribosomal biogenesis
RNR	Ribonucleotide reductase
RP	Ribosomal protein
TLS	Translesion synthesis
TOR	Target of rapamycin
ZIP	Zero interaction potency
