# Supplementary material for: Yeast Phenomic Analysis Reveals DNA Repair, pH Homeostasis, and Ribosomal Biogenesis as Modulators of Anticancer Ruthenium Complex KP1019
Source: Int J Mol Sci. 2026 Apr 4;27(7):3275. doi: 10.3390/ijms27073275 (PMC13073065; doi:10.3390/ijms27073275)

Supplemental File 1. Representative cell array images indicating the data selected for analysis.

On page 2, the table at left indicates which cell arrays were analyzed and the representative images are numbered according to the table in subsequent slides. Cell arrays that were removed for data quality reasons are shown with the red 'X' mark on them.

On pages 3–8 are representative images that are annotated in accord with the table on page 2. The complete set of images that was analyzed (i.e., the complete time series for all images) is available upon request.

Overview

scan	plate	MP	[KP1019]	plate#
1	1	RF1	0	1
1	2	RF1	10	2
1	3	RF1	20	3
1	4	mp13	0	
1	5	mp13	10	
1	6	mp13	20	
1	7	mp1	0	
1	8	mp1	10	
1	9	mp1	20	
1	10	mp2	0	
2	1	mp2	10	
2	2	mp2	20	
2	3	mp3	0	4
2	4	mp3	10	5
2	5	mp3	20	6
2	6	mp4	0	7
2	7	mp4	10	8
2	8	mp4	20	9
2	9	mp5	0	10
2	10	mp5	10	11
3	1	mp5	20	12
3	2	mp6	0	13
3	3	mp6	10	14
3	4	mp6	20	15
3	5	mp7	0	16
3	6	mp7	10	17
3	7	mp7	20	18
3	8	mp8	0	19
3	9	mp8	10	20
3	10	mp8	20	21
4	1	mp9	0	22
4	2	mp9	10	23
4	3	mp9	20	24
4	4	mp10	0	25
4	5	mp10	10	26
4	6	mp10	20	27
4	7	mp11	0	28
4	8	mp11	10	29
4	9	mp11	20	30
4	10	mp12	0	31
5	1	mp12	10	32
5	2	mp12	20	33
5	3	mp14	0	34
5	4	mp14	10	35
5	5	mp14	20	36
5	6	mp15	0	37
5	7	mp15	10	38
5	8	mp15	20	39
5	9	RF2	0	40
5	10	RF2	10	41
6	1	RF2	20	42
6	2	mp1	0	43
6	3	mp1	10	44
6	4	mp1	20	45
6	5	mp2	0	46
6	6	mp2	10	47
6	7	mp2	20	48

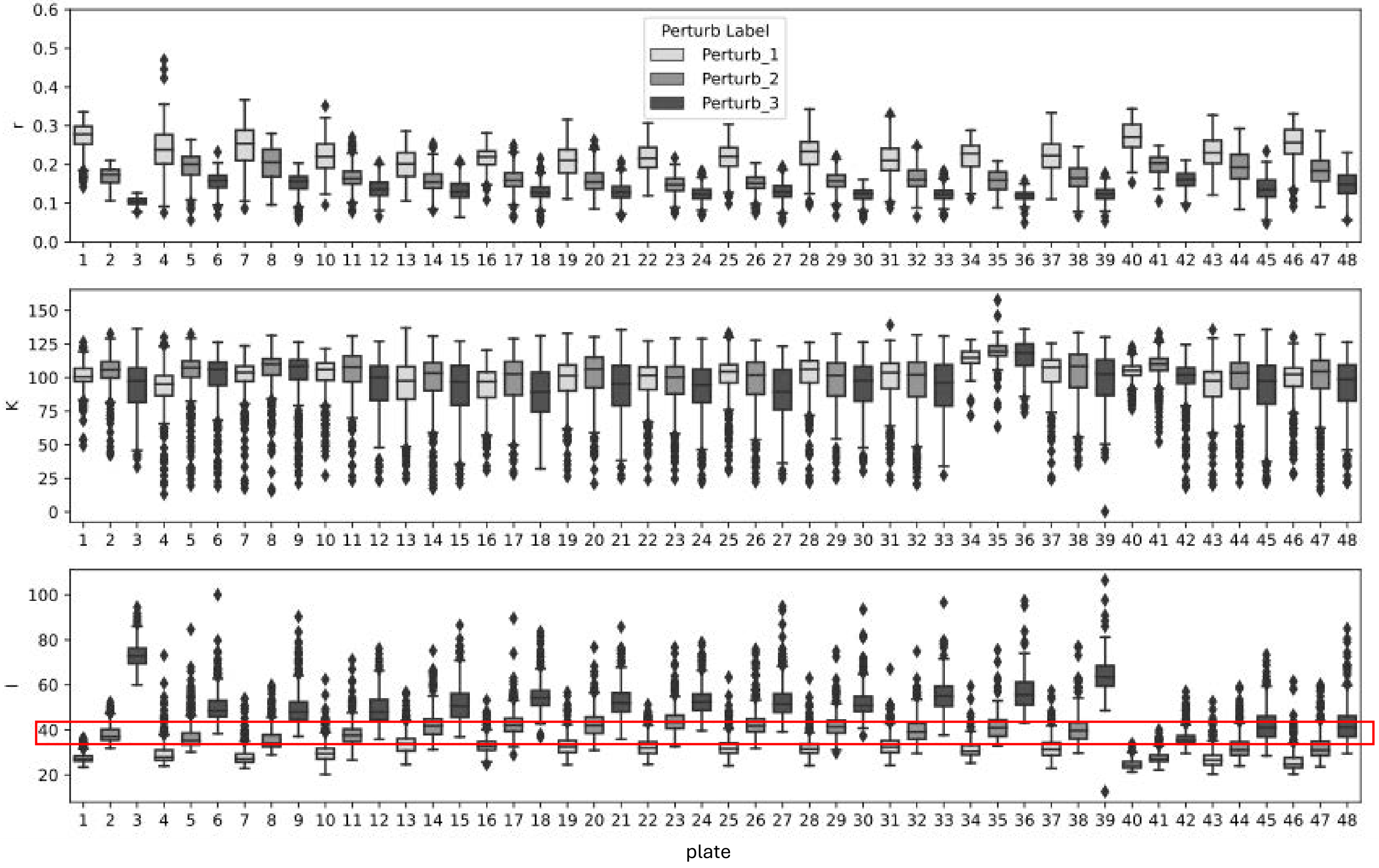

41HRs
scan1

RF1: 1-3

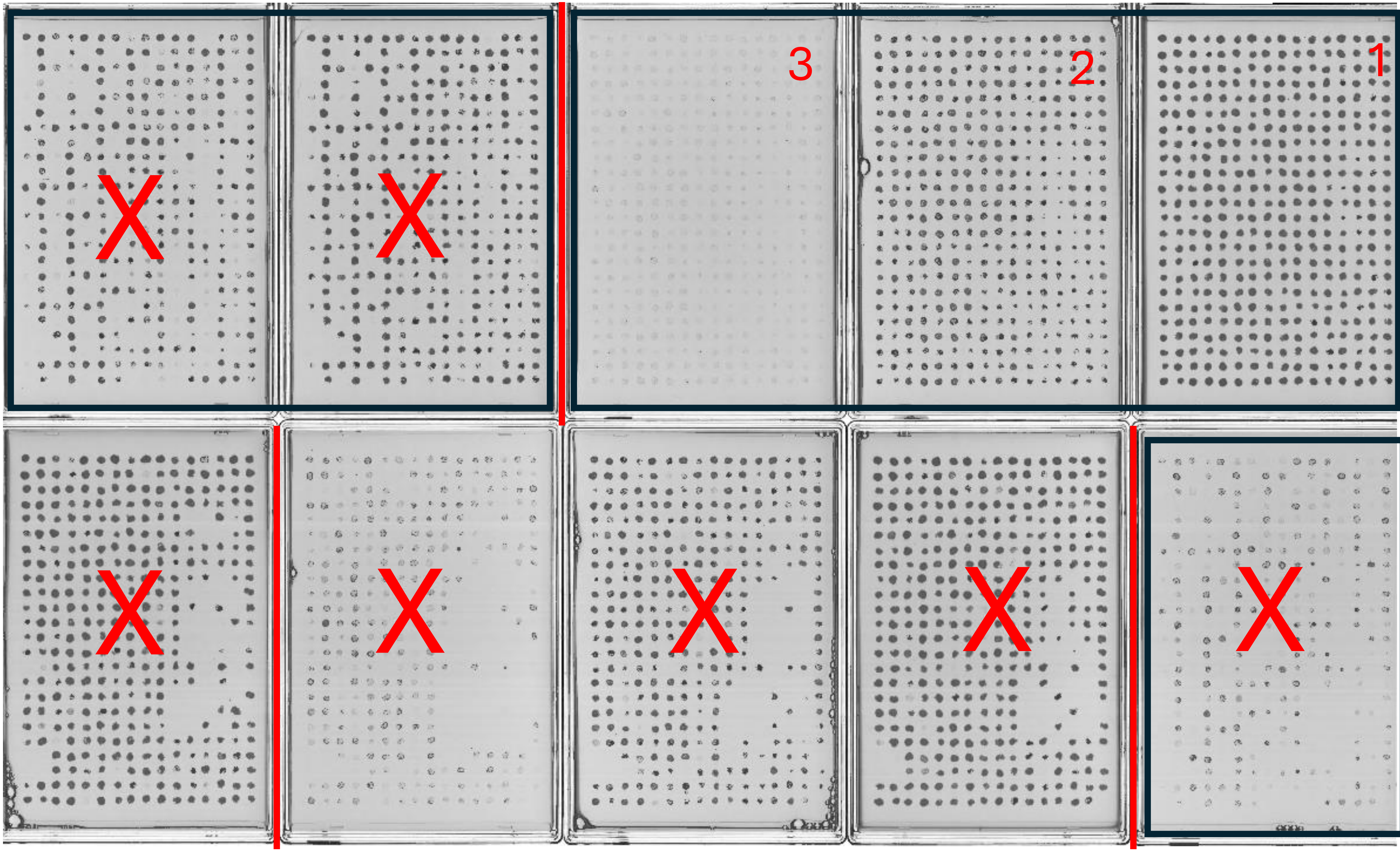

41HRs
scan2

MP3 (4-6), MP4 (7-9), MP5 (10,11)

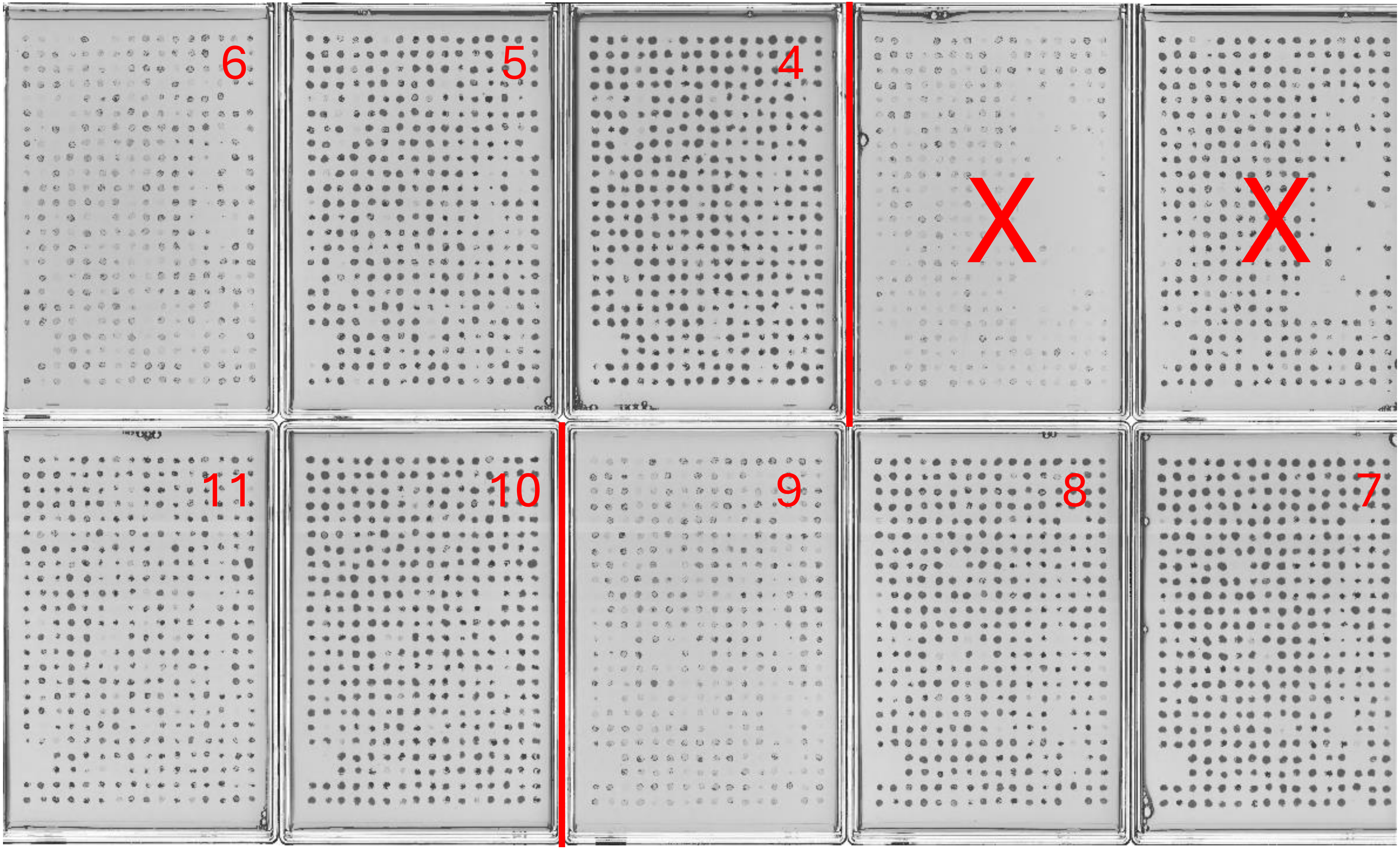

41HRs
scan3

MP5 (12), MP6 (13-15), MP7 (16-18), M P8 (19-21)

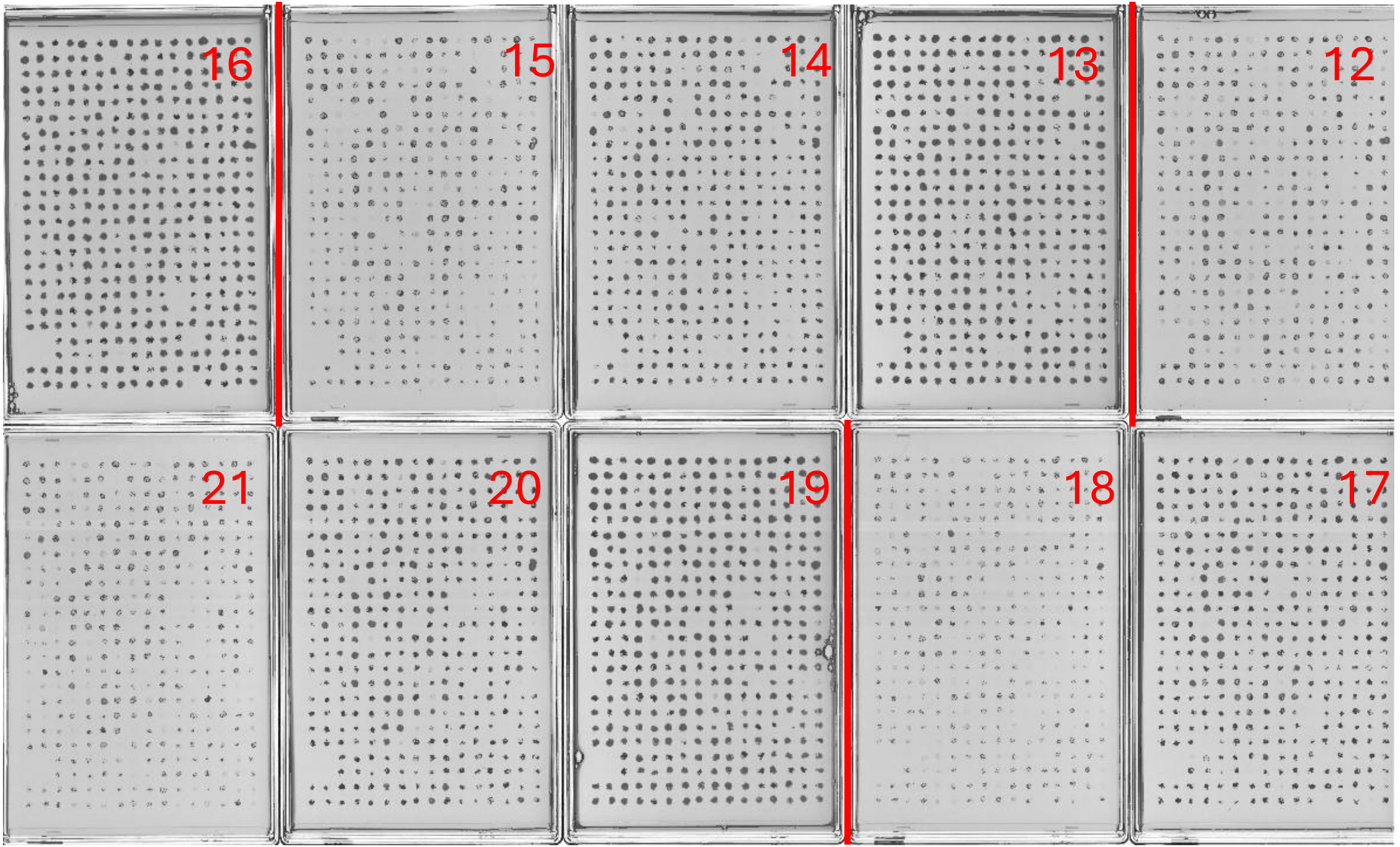

41HRs
scan4

MP9 (22-24), MP10 (25-27, MP11 (28-30), MP12 (31)

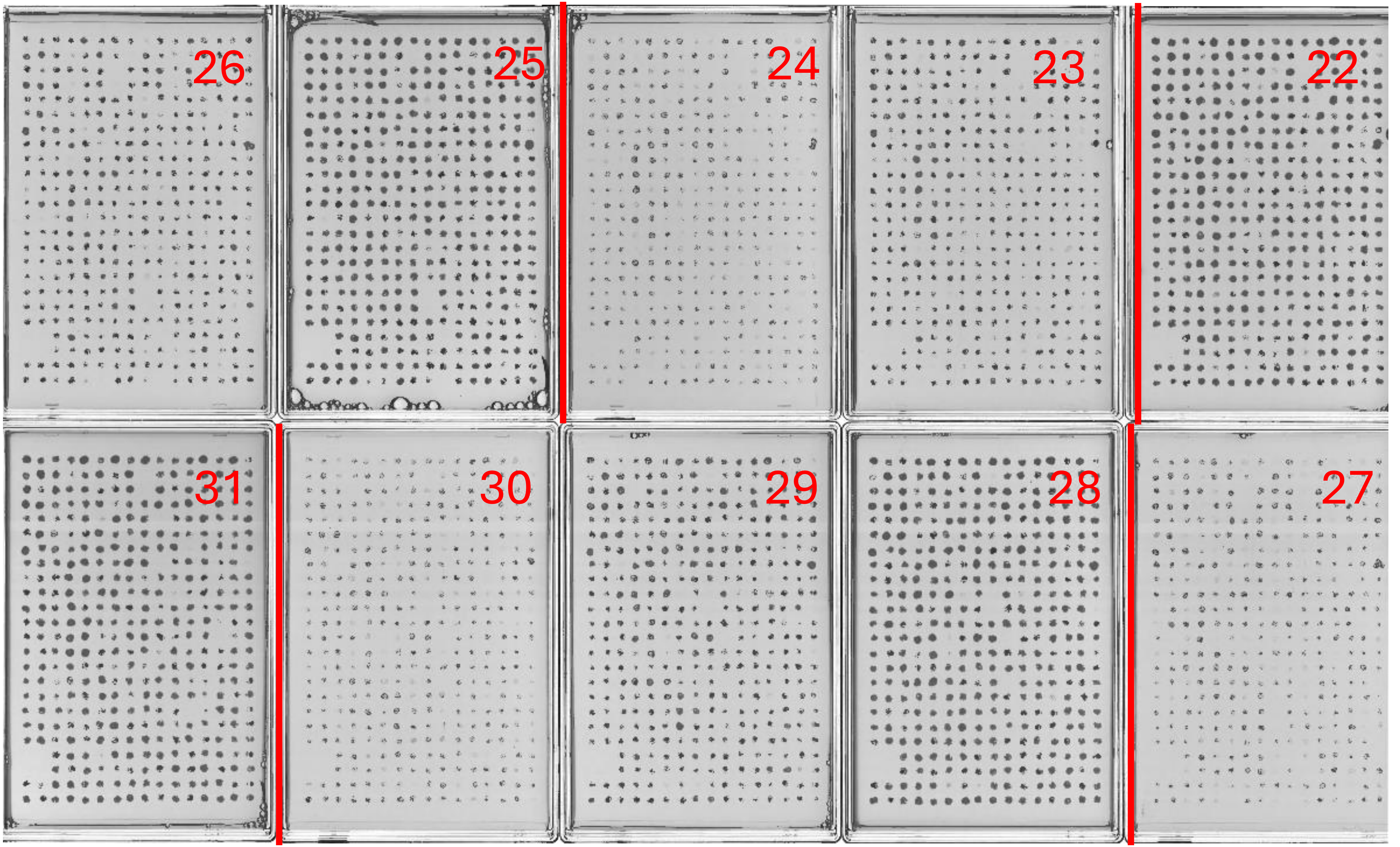

41HRs
scan5

MP12 (32, 33) , MP14 (34-36), MP15 (37-39), RF2 (40,41)

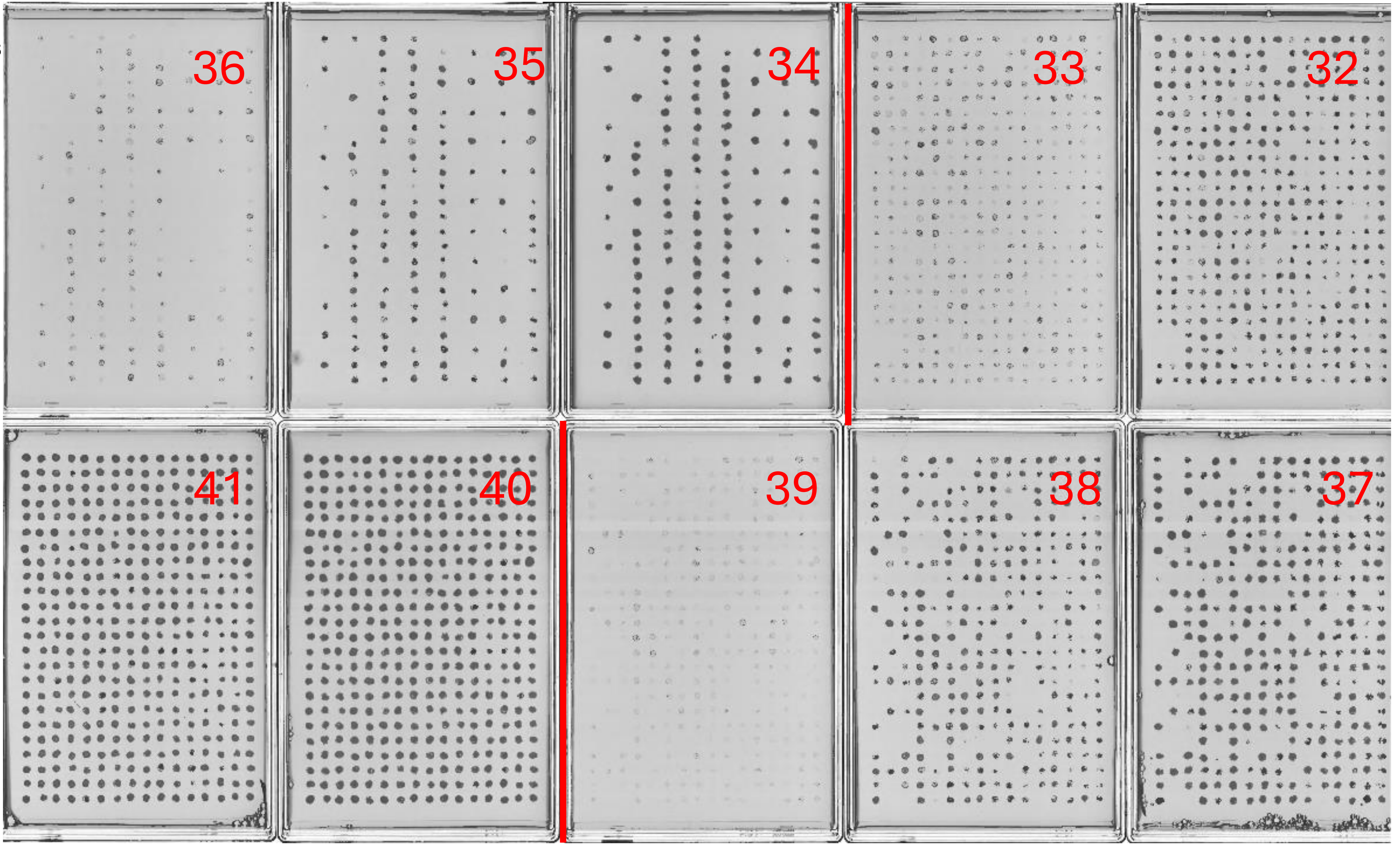

41HRs
Scan6

RF2 (42) , MP1 (43-45), MP2 (46-48)

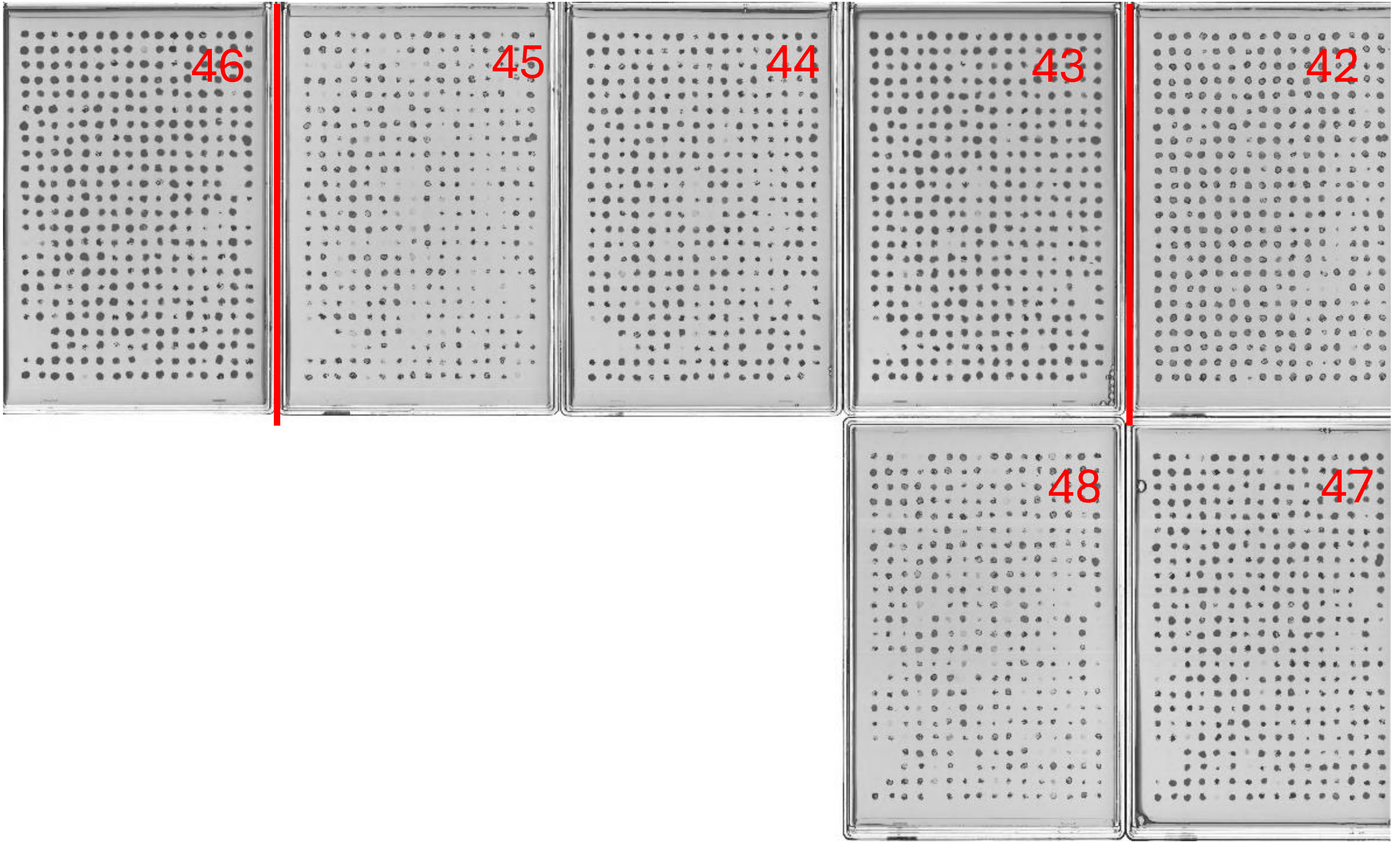

Supplement: Supplementary file 1 [file ijms-27-03275-s001.zip › supplemental files revised/S1_Representative_Cell_Array_Images.pdf]
