# Supplementary material for: Yeast Phenomic Analysis Reveals DNA Repair, pH Homeostasis, and Ribosomal Biogenesis as Modulators of Anticancer Ruthenium Complex KP1019
Source: Int J Mol Sci. 2026 Apr 4;27(7):3275. doi: 10.3390/ijms27073275 (PMC13073065; doi:10.3390/ijms27073275)
Supplement: Supplementary file 1 [file ijms-27-03275-s001.zip › supplemental files revised/S2_Supplemental Figures.pdf]

Supplemental File 2: Supplemental Figures

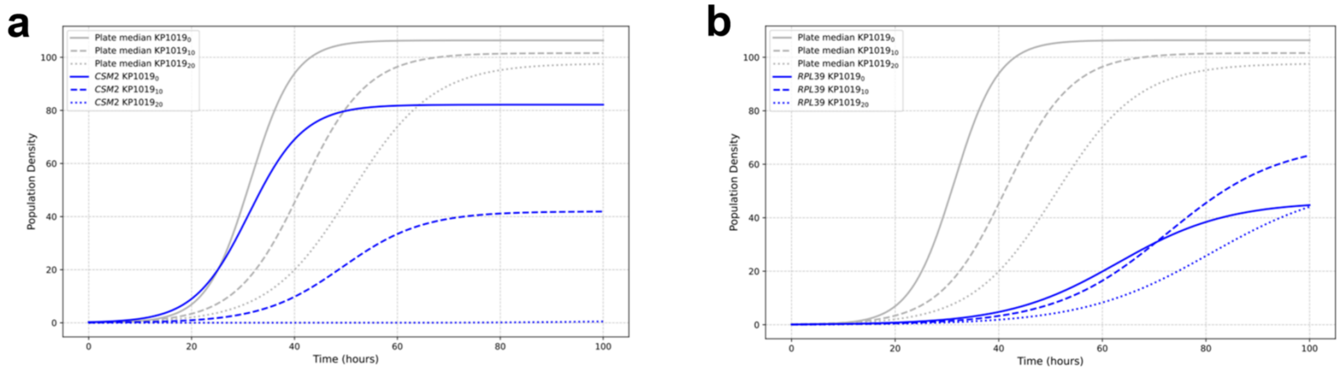

Supplemental Figure 1. Examples of growth curves for KP1019 enhancing and suppressing mutations. For both graphs, gray lines represent the median growth curve for the entire plate containing 384 yeast strains and blue lines represent the growth curves for yeast lacking (**a**) *CSM2* or (**b**) *RPL39*. Solid lines correspond to growth in the absence of the drug. Dashed and dotted lines correspond to growth in the presence of 10 and 20 μg/mL KP1019, respectively. (**a**) Loss of *CSM2* enhances the effects of KP1019 on both *K* and *L*. (**b**) Loss of *RPL39* decreases *K*, increases *L*, and suppresses the effects of KP1019 on both parameters.

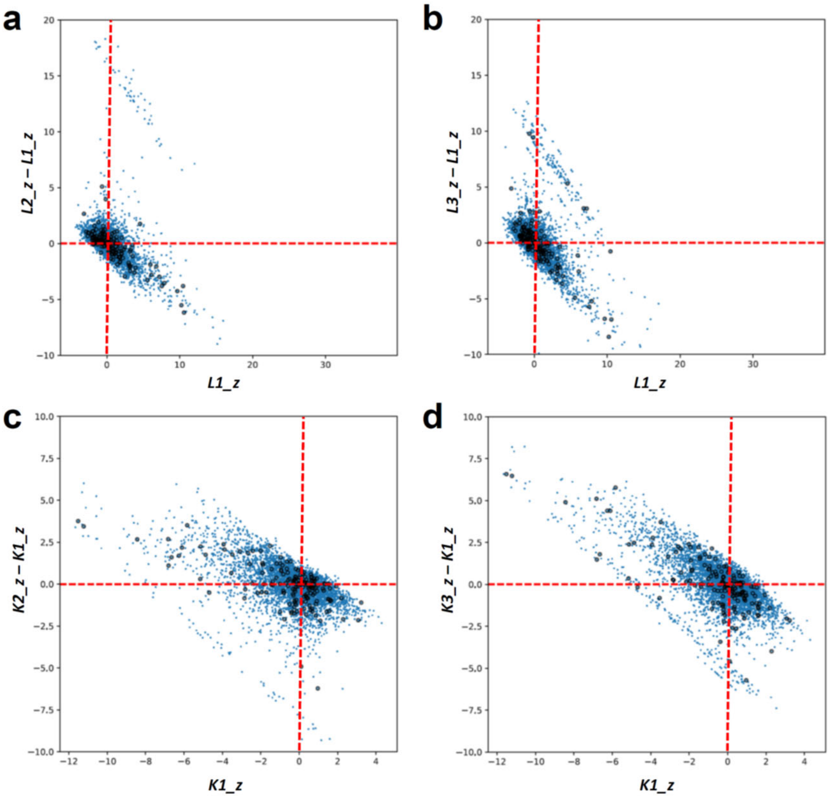

e

low intrinsic fitness (L1_z > 0.5)			
ΔCPP	RP (n = 130)	Non-RP (n = 1756)	p-value
K2 -K1	2.89	1.65	5.04×10^{-2}
K3-K1	-3.8	-7.01	**6.32×10^{-3}**
L2-L1	7.06	8.39	**3.85×10^{-4}**
L3-L1	18	21	**1.58×10^{-4}**

normal intrinsic fitness (-0.5 < L1_z < 0.5)			
ΔCPP	RP (n = 21)	Non-RP (n = 1264)	p-value
K2 -K1	5.3	2.77	1.96×10^{-1}
K3-K1	-10.7	-4.3	8.16×10^{-1}
L2-L1	8.09	8.12	8.32×10^{-1}
L3-L1	20.1	20.1	4.00×10^{-1}

high intrinsic fitness (L1_z < -0.5)			
ΔCPP	RP (n = 24)	Non-RP (n = 1756)	p-value
K2 -K1	3.42	3.83	6.16×10^{-1}
K3-K1	-2.51	-0.749	9.45×10^{-1}
L2-L1	6.29	7.82	4.03×10^{-1}
L3-L1	16.1	19.3	1.53×10^{-1}

Supplemental Figure 2. Suppression of KP1019 activity in ribosomal protein (RP) mutants exceeds that predicted by reduced fitness alone. (**a–d**) Adjusted CPP z-scores (CPP_z_drug – CPP_z_nodrug) for 10 μg/mL (**a**,**c**) and 20 μg/mL KP1019 (**b**,**d**) were plotted as a function of the corresponding CPP z-score in the absence of KP1019. These plots reveal the trend that low intrinsic fitness (low growth in the absence of drug, as indicated by negative *K1* z-scores or positive *L1* z-scores) tends to correlate with suppression of KP1019 activity, as indicated by positive adjusted *K* z-scores (*K2_z - K1_z* or *K3_z - K1_z*) or negative adjusted *L* z-scores (*L2_z - L1_z* or *L3_z - L1_z*). Ribosomal protein deletion strains, which are circled in black within the scatter plots, trend toward drug suppression. Though all RP deletion strains are highlighted in these graphs, not all RP deletion strains were identified as KP1019 resistant. (**e**) To mitigate the impact of intrinsic fitness, RP and non-RP strains were disaggregated into three groups with low, normal, or high fitness as defined by *L* z-score in the absence of KP1019 (*L1_z*). The median ΔCPP (CPP_drug – CPP_no_drug) is provided for each group. t-tests compared ΔCPP values for RP and non-RP deletions. Statistically significant differences ($p < 0.05$) are in bold. Combined, these data support the conclusion that although low intrinsic fitness correlates with KP1019 resistance for all strains, RP deletions that cause lower intrinsic fitness have disproportionately strong impacts on KP1019 resistance.

a

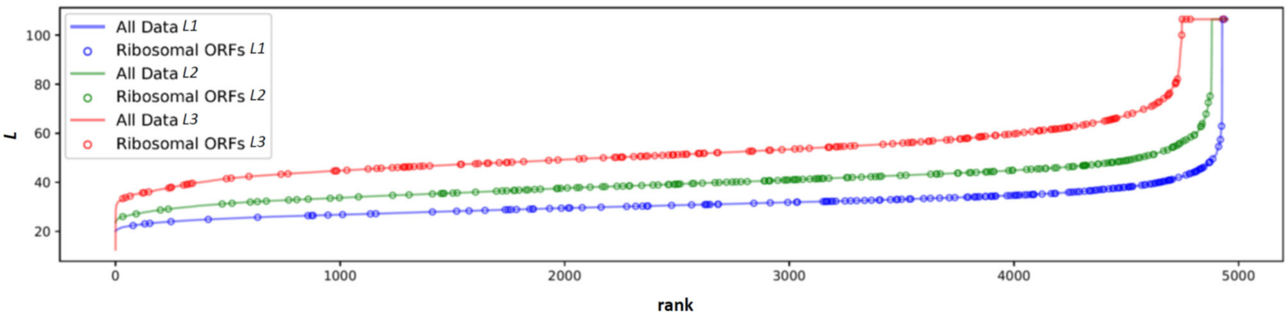

b

KP1019 (μg/mL)	RP median *L* z-score	RP MAD	Control median *L* z-score	Control MAD	p-value
0	1.9	2.3	1.8	2.3	**3.63E-02**
10	0.77	1.4	1	1.6	**9.20E-03**
20	0.56	1.5	0.99	1.6	**1.46E-03**

c

KP1019 (μg/mL)	RP median *K* z-score	RP MAD	Control median *K* z-score	Control MAD	p-value
0	-0.75	2	-1.3	2.4	9.89E-01
10	-0.57	1.4	-0.9	1.9	1.53E-01
20	-0.29	1.2	-0.85	1.6	**1.12E-02**

Supplemental Figure 3. Rank-based and nearest neighbor analyses of KP1019 suppression by RP gene deletion. To determine whether suppression of KP1019 activity in RP deletion strains was greater than would be predicted based on low fitness alone, the CPPs *L* (**a**,**b**) and *K* (**c**) were analyzed. (**a**) First all tested deletion strains were ordered from lowest to highest *L* value at each KP1019 concentration; these data were graphed with blue corresponding to 0 μg/mL, green corresponding to 10 μg/mL, and red corresponding to 20 μg/mL. Positions of RP deletion strains are highlighted with circles. As KP1019 concentration increases, the distribution of RP deletion strains shifts left and downward along the curve, indicating higher rank, which corresponds with greater suppression of KP1019's impact on *L*. Mann-Whitney U tests comparing RP deletion strain rank in the presence vs. absence of KP1019 revealed statistically significant differences between 0 and 10 μg/mL ($p = 2.29 \times 10^{-3}$) and between 0 and 20 μg/mL ($p = 2.31 \times 10^{-6}$). (**b**,**c**) Next all tested strains were ordered based on *L* z-score in the absence of KP1019. Within this ranked list, each RP deletion strain was identified. The non-RP deletion strains immediately after each RP deletion strain were selected as controls with similar intrinsic fitness (growth in the absence of KP1019). The median *L* (**b**) and *K* (**c**) z-scores for each group of strains (RP deletions and non-RP controls) are presented, as are the median absolute deviation (MAD) and results of Mann-Whitney U tests. (**b**) As expected, given the selection strategy for control strains, the RP deletion strains had similar median *L* z-scores in the absence of drug. When *L* z-scores in the presence of KP1019 are compared, the RP deletion strains had significantly lower z-scores than the non-RP controls, indicating that in the presence of drug, the RP deletion strains have higher fitness than their growth-matched controls. (**c**) At 0 and 10 μg/mL KP1019 there was not a significant difference in *K* z-scores between groups. However, at 20 μg/mL KP1019, the RP deletion strains had significantly higher *K* z-scores than the non-RP controls, indicating that the RP deletion strains have higher fitness than their growth-matched controls at that concentration of drug. These results support the model that RP deletion increases resistance to KP1019 in yeast.

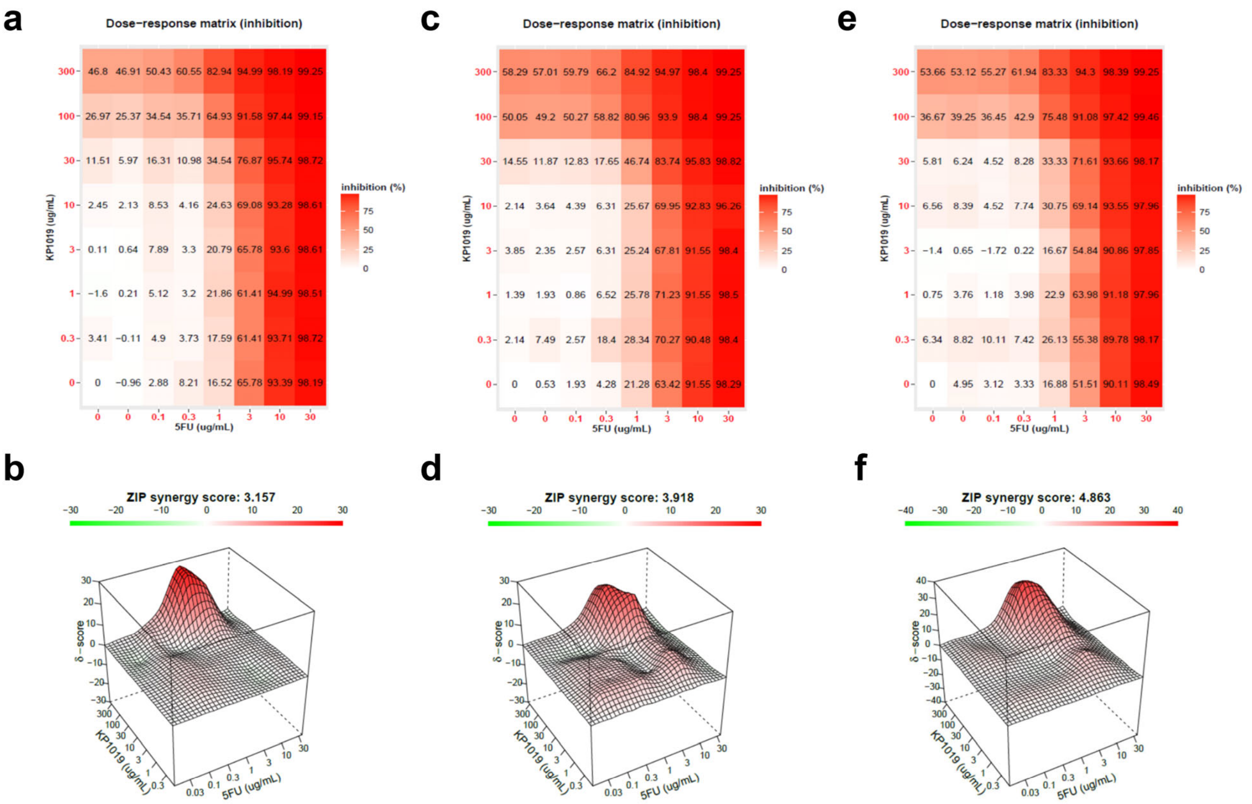

Supplemental Figure 4. Individual trials of KP1019-5-FU interaction assay. Exponentially growing wild-type yeast were treated with varying concentrations of KP1019 for 3h prior to 18-24 hr of growth in the presence of 5-FU. The response matrices (**a**,**c**,**e**) and interaction landscapes (**b**,**d**,**f**) for three trials are included. (**a**,**b**) Trial 1. (**c**,**d**) Trial 2. (**e**,**f**) Trial 3.

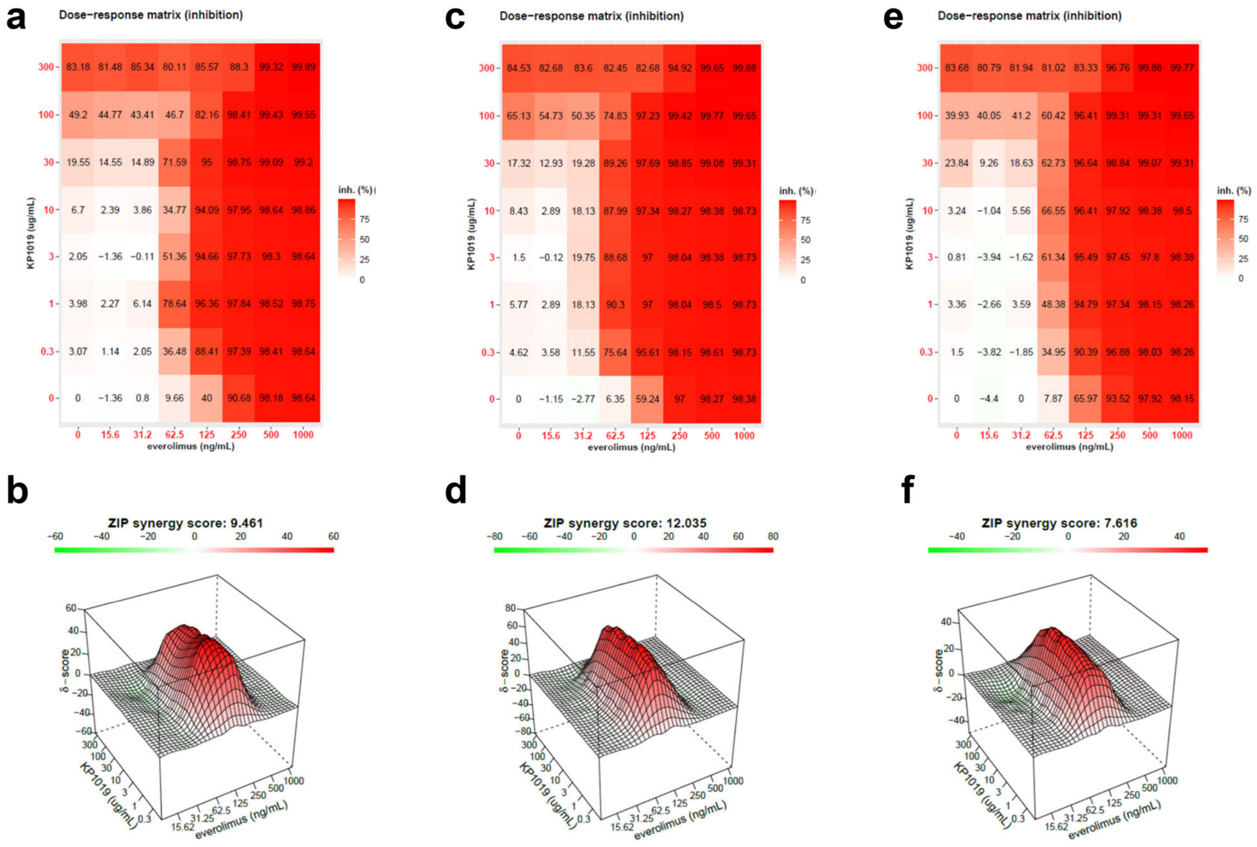

Supplemental Figure 5. Individual trials of KP1019-everolimus interaction assay. Exponentially growing wild-type yeast were treated with varying concentrations of KP1019 for 3h prior to 18-24 hr of growth in the presence of everolimus. The response matrices (**a**,**c**,**e**) and interaction landscapes (**b**,**d**,**f**) for three trials are included. (**a**,**b**) Trial 1. (**c**,**d**) Trial 2. (**e**,**f**) Trial 3.
