# Supplementary material for: Yeast Phenomic Analysis Reveals DNA Repair, pH Homeostasis, and Ribosomal Biogenesis as Modulators of Anticancer Ruthenium Complex KP1019
Source: Int J Mol Sci. 2026 Apr 4;27(7):3275. doi: 10.3390/ijms27073275 (PMC13073065; doi:10.3390/ijms27073275)

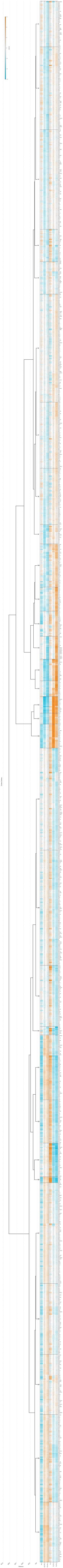

Supplement: Supplementary file 1 [file S4_Hierarchical_Clustering.pdf › supplemental files revised/S4_Hierarchical_Clustering.pdf]
